# Supplementary material for: Does family planning counselling during health service contact improve postpartum modern contraceptive uptake in Ethiopia? A nationwide cross-sectional study
Source: BMJ Open. 2022 May 10;12(5):e060308. doi: 10.1136/bmjopen-2021-060308 (PMC9092163; doi:10.1136/bmjopen-2021-060308)
Supplement: Supplementary data [file bmjopen-2021-060308supp001.pdf]

Table S1; List of covariates and their definition

Variable	Definition
Household wealth quintile	The household wealth index was computed by principal component analysis (PCA) using the household's ownership of selected items, such as radio and television, and possession of household animals, including oxen, cow, camel; and materials used for housing construction, and types of water access and sanitation facilities taking into consideration of urban-rural difference. We categorised the wealth index score into three equal parts labelled as lowest, middle, and highest
Residence	Participant place of residence coded as urban and rural. Urban was defined as a locality with 2,000 or more inhabitants. Moreover, all administrative capitals, including Region, Zone and Woreda, and localities in which urban dwellers' associations were established were considered urban residences, irrespective of the population size. Rural residences comprise all areas not classified as urban.
Region	Ethiopia has nine regional states and two city administrations. For this study purpose, we merged the emerging regional states of Ethiopia, such as Afar, Somali, Benishangul-Gumuz, Harari and Dire Dawa, into one group as others. Hence, the region categorised into Tigray, Amhara, Oromia, SNNP, Addis Ababa and others
Women's age	Women's age was grouped into <25 years, 25-29 years, and ≥30 years
Women's educational status	Women's education status is available in EDHS coded as no education, primary, secondary, and higher. Since the frequency of women who had a higher level of education was low, we merged the secondary and higher into 'secondary and above.'
Women's employment	Participants were grouped as employed when they reported as working within seven days preceding the survey, including women who did not work in the past seven days but who are regularly employed and were absent from work for leave, illness, vacation, or any other such reason, otherwise as not employed
Marital status	The marital status was categorised as married/in union, including married and living with a partner, and not married/in union, including never in union, widowed, divorced, and no longer living together/separated
Women's religion	Women's religion is available in the EDHS coded as Orthodox, Muslim, Protestant, Catholic, Traditional, and other. We merged Orthodox, Protestant and Catholic into Christian and Traditional and

	others into other. Hence, religion in this study was categorised as Christian, Muslim and Others
Parity	Parity of the participant women was categorised into primiparity (para 1), multiparity (para 2-4) and grand multiparity (para ≥5)
Wantdeness of the last child	Wantdeness of the last child grouped into planned, unplanned, and unwanted
Sex of last-child	Sex of the last child was categorised as male or female
Survival status of the last child	Survival status of the last child categorised as dead and alive
ANC for the pregnancy of the last birth	ANC visit at least once during the pregnancy of last birth was coded as yes, if not as no
Place of delivery for the last birth	Place of delivery was labelled as home if delivered at their home or any other home, and health facility if delivered in any one of the health facilities- public, private, or non-governmental health facility.
PNC following the last birth	PNC was categorised as yes when they received any checking their health status either at a health facility or at home within six weeks following the last birth; otherwise, no
Postpartum period/interview time	The postpartum period is the time between the birth of the last child and the interview date and was categorised into 0-6 months and 7-12 months
Future fertility intention	Future fertility intention was categorised as have more, no more and undecided
Receiving family planning messages from media	receiving of family planning messages from media was labelled as 'yes' when women received any family planning messages from any one of the following: radio, television, newspaper/magazine or text message on mobile phone in the past few months preceding the survey, and 'no' if did not receive any message from any one of the media channels.
Distance to a health facility for getting medical help	The distance to a health facility for getting medical help was captured by a self-reported response as a big problem vs not a big problem
Health insurance	Health insurance was categorised as yes and no, corresponding for having and not having health insurance, respectively.
