# Supplementary material for: Does family planning counselling during health service contact improve postpartum modern contraceptive uptake in Ethiopia? A nationwide cross-sectional study
Source: BMJ Open. 2022 May 10;12(5):e060308. doi: 10.1136/bmjopen-2021-060308 (PMC9092163; doi:10.1136/bmjopen-2021-060308)
Supplement: Supplementary data [file bmjopen-2021-060308supp002.pdf]

Table S2. Sample size for the adjusted and unadjusted models among all and women stratified by place of delivery, PNC visits and interview time postpartum in Ethiopia 2016

Samples		**Unadjusted**	**Adjusted**
All women		1,650	1,550
Place of delivery	Home delivery	873	831
	Facility delivery	743	689
PNC visits	Had PNC visits	423	394
	Had no PNC visits	1,203	1,134
Interview time postpartum	0-6 months postpartum	934	888
	7-12 months postpartum	716	662
