# Supplementary material for: Does family planning counselling during health service contact improve postpartum modern contraceptive uptake in Ethiopia? A nationwide cross-sectional study
Source: BMJ Open. 2022 May 10;12(5):e060308. doi: 10.1136/bmjopen-2021-060308 (PMC9092163; doi:10.1136/bmjopen-2021-060308)
Supplement: Supplementary data [file bmjopen-2021-060308supp003.pdf]

Table S3. Sociodemographic characteristics, reproductive histories, and health service utilisations of the study participants in Ethiopia 2016 (n=1,650)

| Variable | Category | Frequency | Percentage |
|---|---|---|---|
| Household wealth index | Lowest | 317 | 19.2 |
| | Middle | 685 | 41.5 |
| | Highest | 647 | 39.2 |
| Residence | Urban | 255 | 15.4 |
| | Rural | 1,395 | 84.6 |
| Region | Tigray | 160 | 9.7 |
| | Amhara | 363 | 22.0 |
| | Oromia | 642 | 38.9 |
| | SNNP | 340 | 20.6 |
| | Addis Ababa | 55 | 3.3 |
| | Others | 89 | 5.4 |
| Women's age at birth of the last child | <25 years | 634 | 38.4 |
| | 25-29 years | 456 | 27.7 |
| | ≥30 years | 559 | 33.9 |
| Women's educational level | No education | 881 | 53.4 |
| | Primary | 586 | 35.5 |
| | Secondary and above | 183 | 11.1 |
| Marital status | Not married/in union | 73 | 4.4 |
| | Married/in union | 1,577 | 95.6 |
| Women's current job | Not working | 996 | 60.4 |
| | Working | 654 | 39.6 |
| Religion | Christian | 981 | 59.5 |
| | Muslim | 635 | 38.5 |
| | Others | 33 | 2.0 |
| Women's decision-making autonomy | No autonomy | 570 | 36.6 |
| | Having autonomy | 987 | 63.4 |
| Parity | Primiparity | 409 | 24.8 |
| | Multiparity | 850 | 51.5 |
| | Grand multiparity | 390 | 23.7 |
| Wantdeness of the last pregnancy | Unintended | 449 | 27.2 |
| | Intended | 1,200 | 72.8 |
| Sex of the last child | Male | 822 | 49.8 |
| | Female | 828 | 50.2 |
| Survival status of the last child | Died | 41 | 2.5 |
| | Alive | 1,608 | 97.5 |
| ANC visits for pregnancy of the last birth | No | 346 | 21.0 |
| | Yes | 1,302 | 79.0 |
| Place of delivery for the last birth | Home | 873 | 54.0 |
| | Health facility | 744 | 46.0 |
| PNC visits following the last birth | No | 1,203 | 76.1 |
| | Yes | 379 | 23.9 |
| Postpartum period/ Interview time | 0-6 moths | 934 | 56.6 |
| | 7-12 months | 716 | 43.4 |
| Fertility intention | No more | 629 | 38.2 |
| | Have another | 1,017 | 61.8 |
| receiving of family planning messages from media | No | 1,152 | 69.8 |
| | Yes | 498 | 30.2 |
| Distance to a health facility | A big problem | 911 | 55.2 |
| | Not a big problem | 739 | 44.8 |
| Have health insurance | No | 1,571 | 95.2 |
| | Yes | 78 | 4.8 |
| Family planning counselling | Not counselled | 856 | 51.9 |
| | Counselled | 794 | 48.1 |
