# Supplementary material for: Does family planning counselling during health service contact improve postpartum modern contraceptive uptake in Ethiopia? A nationwide cross-sectional study
Source: BMJ Open. 2022 May 10;12(5):e060308. doi: 10.1136/bmjopen-2021-060308 (PMC9092163; doi:10.1136/bmjopen-2021-060308)
Supplement: Author's manuscript [file bmjopen-2021-060308.draft_revisions.pdf]

BMJ Open

BMJ Open is committed to open peer review. As part of this commitment we make the peer review history of every article we publish publicly available.

When an article is published we post the peer reviewers' comments and the authors' responses online. We also post the versions of the paper that were used during peer review. These are the versions that the peer review comments apply to.

The versions of the paper that follow are the versions that were submitted during the peer review process. They are not the versions of record or the final published versions. They should not be cited or distributed as the published version of this manuscript.

BMJ Open is an open access journal and the full, final, typeset and author-corrected version of record of the manuscript is available on our site with no access controls, subscription charges or pay-per-view fees (http://bmjopen.bmj.com).

If you have any questions on BMJ Open's open peer review process please email info.bmjopen@bmj.com

BMJ Open

Family planning counselling and postpartum contraceptive uptake in Ethiopia

Journal:	*BMJ Open*
Manuscript ID	bmjopen-2021-060308
Article Type:	Original research
Date Submitted by the Author:	18-Dec-2021
Complete List of Authors:	Mruts, Kalayu Brhane; Curtin University, School of Population Health<br>Tessema, Gizachew; Curtin University, School of Population Health<br>Dunne, Jennifer; Curtin University, School of Population Health<br>Gebremedhin, Amanuel Tesfay; Curtin University, School of Population Health; Wesfarmers Centre for Vaccine and Infectious Disease, Telethon Kids Institute, University of Western Australia, Perth, Australia<br>Scott, Jane; Curtin University, School of Population Health<br>Pereira, G.F; Curtin University, School of Population Health; Centre for Fertility and Health (CeFH), Norwegian Institute of Public Health, School of Population
Keywords:	PUBLIC HEALTH, EPIDEMIOLOGY, Reproductive medicine < GYNAECOLOGY

SCHOLARONE™
Manuscripts

I, the Submitting Author has the right to grant and does grant on behalf of all authors of the Work (as defined in the below author licence), an exclusive licence and/or a non-exclusive licence for contributions from authors who are: i) UK Crown employees; ii) where BMJ has agreed a CC-BY licence shall apply, and/or iii) in accordance with the terms applicable for US Federal Government officers or employees acting as part of their official duties; on a worldwide, perpetual, irrevocable, royalty-free basis to BMJ Publishing Group Ltd ("BMJ") its licensees and where the relevant Journal is co-owned by BMJ to the co-owners of the Journal, to publish the Work in this journal and any other BMJ products and to exploit all rights, as set out in our licence.

The Submitting Author accepts and understands that any supply made under these terms is made by BMJ to the Submitting Author unless you are acting as an employee on behalf of your employer or a postgraduate student of an affiliated institution which is paying any applicable article publishing charge ("APC") for Open Access articles. Where the Submitting Author wishes to make the Work available on an Open Access basis (and intends to pay the relevant APC), the terms of reuse of such Open Access shall be governed by a Creative Commons licence – details of these licences and which Creative Commons licence will apply to this Work are set out in our licence referred to above.

Other than as permitted in any relevant BMJ Author's Self Archiving Policies, I confirm this Work has not been accepted for publication elsewhere, is not being considered for publication elsewhere and does not duplicate material already published. I confirm all authors consent to publication of this Work and authorise the granting of this licence.

For peer review only

Family planning counselling and postpartum contraceptive uptake in Ethiopia

Kalayu Brhane Mruts[1 *], Gizachew A Tessema[1,2], Jennifer Dunne[1], Amanuel Tesfay Gebremedhin[1,3], Jane A. Scott[1], Gavin Pereira[1,4,5]

[1] School of Population Health, Curtin University, Perth, Australia

[2] School of Public Health, University of Adelaide, Adelaide, Australia

[3] Wesfarmers Centre for Vaccine and Infectious Disease, Telethon Kids Institute, University of Western Australia, Perth, Australia

[4] Centre for Fertility and Health (CeFH), Norwegian Institute of Public Health, Oslo, Norway

[5]enAble Institute, Curtin University, Perth Australia

[*] **Corresponding Author:**

Kalayu Brhane Mruts

Curtin School of Population Health, Curtin University

GPO Box U1987, Perth Western Australia 6845

Email: kalayu.brhanemruts@postgrad.curtin.edu.au

Phone: +61 478014036

For peer review only

Word count: 2,932

For peer review only - http://bmjopen.bmj.com/site/about/guidelines.xhtml

ABSTRACT

Objective: Women in their postpartum period are vulnerable to unintended pregnancy and may benefit from family planning counselling. Previous research from Ethiopia have inconsistent findings in the association between family planning counselling and postpartum modern contraceptive uptake. This study examined the association between family planning counselling receipt during the 12 months preceding the survey and postpartum modern contraceptive uptake

Design: A cross-sectional study extracted from the Ethiopian Demographic and Health survey conducted in 2016

Setting: Ethiopia

Participants: 1650 women in the postpartum period who had contact with service delivery points during the 12 months preceding the survey

Primary outcome: We estimated an adjusted relative risk of postpartum modern contraceptive uptake

Results: Approximately half of the women have missed the opportunity to receive family planning counselling at the health service contact points during the 12 months preceding the survey. The postpartum modern contraceptive uptake was 27%. Compared to women who did not receive counselling for family planning, women who received counselling had higher contraceptive uptake (RR 1.32, 95% CI 1.04, 1.67).

Conclusion: Significant numbers of women have missed the opportunity of receiving family planning counselling during contact with health service delivery points. Modern contraceptive uptake among postpartum women was low. Despite this, our findings revealed that family planning counselling was associated with improved postpartum modern contraceptive uptake.

Keywords: Contraceptive uptake, postpartum, family planning counselling, modern contraceptive, Ethiopia

For peer review only

For peer review only - http://bmjopen.bmj.com/site/about/guidelines.xhtml

Article Summary

Strengths and limitations of this study

- This study used a large national survey data to examine the association between family planning counselling and postpartum modern contraceptive uptake
- The family planning counselling and contraceptive uptake were measured using self-reported with no available previous counselling information that might result in recall bias
- This is a cross-sectional study that could not assure the causal relationship between family planning counselling and postpartum modern contraceptive uptake

For peer review only

For peer review only - http://bmjopen.bmj.com/site/about/guidelines.xhtml

INTRODUCTION

Adopting modern contraceptive methods can improve maternal, neonatal, and infant health outcomes [1]. The postpartum period, defined as the first 12 months following childbirth, is the most critical time for women to adopt modern contraceptive methods. Family planning counselling provided during pregnancy, childbirth, and postpartum periods has been shown to improve modern contraceptive uptake [2]. A delay in modern contraceptive uptake in the postpartum period is highly likely to result in unintended and closely spaced pregnancies [1]. Countries in sub-Saharan Africa (SSA) have the highest number of unintended and closely spaced pregnancies [3 4], accounting for around 68% and 42% of global maternal and neonatal mortality [5 6]. Unintended and closely spaced pregnancies are public health issues strongly associated with increased adverse health consequences for the mother and birth outcomes, such as preterm birth, stillbirth, low birth weight and neonatal and infant mortality [7-9].

Although maternal and child mortality has declined in Ethiopia over the last two decades [10], rates of unintended pregnancy remain high, coupled with a high fertility rate and low postpartum modern contraceptive uptake[10-12]. According to the Ethiopian Demographic and Health (EDHS) 2016 report, the incidence of maternal and neonatal mortality are 412 per 100,000 live births and 29 deaths per 1,000 live births, respectively [10]. Moreover, it is reported that a short interbirth interval had been associated with stillbirth, preterm birth, neonatal mortality, and low birth weight in Ethiopia [13-15]. Modern contraceptive uptake during the postpartum period is one of the key strategies that can help prevent unintended and closely spaced pregnancies, thereby improving maternal, neonatal, and infant health outcomes. Furthermore, increasing pregnancy spacing could prevent up to 32% of maternal and 21% of child mortalities [1].

In order to improve postpartum contraceptive uptake in low and middle-income countries, the World Health Organisation (WHO) recommends that women receive family planning

For peer review only - http://bmjopen.bmj.com/site/about/guidelines.xhtml

counselling integrated within maternal and child health services, such as antenatal care (ANC), childbirth, postnatal care (PNC), and well-baby care, immunisation and growth monitoring [16]. To increase access to contraception, the Ethiopian Ministry of Health also recommends that women receive family planning counselling at all the health service contact points, such as health facilities and community settings [17]. The health facilities in the country (public, private, or non-governmental) are expected to provide family planning services integrated with all the health services [17]. Additionally, in the community settings, more than 39,000 health extension workers (HEWs) work across 16,000 villages, mainly in the rural community, to provide health education and counselling on maternal and child health and nutrition, including family planning counselling. Providing appropriate family planning counselling about the potential risks and side effects of modern contraceptive methods is essential for women to avoid misperceptions, increase knowledge, and make informed decisions [18 19].

Despite the strong WHO and Ethiopian Ministry of Health recommendations, Ethiopia lacks research at the national level assessing the association between family planning counselling and postpartum modern contraceptive uptake. Previous studies, conducted with small samples at the district level, have reported mixed evidence [20-23]. This study aims to assess the association between family planning counselling, provided either at the health facility by a health care provider or at the community setting by a family planning field worker and postpartum modern contraceptive uptake in Ethiopia using the most recent national representative EDHS 2016. This study will increase our understanding of the contribution of the family planning counselling provided in Ethiopia on postpartum modern contraceptive uptake. The outcomes of this study will provide decision-makers and program managers with an evidence-based to assess and strengthen the counselling services of family planning. Additionally, it will motivate health care providers to provide appropriate family planning information during the counselling sessions.

For peer review only - http://bmjopen.bmj.com/site/about/guidelines.xhtml

METHODS

Study design and setting

We used cross-sectional data from the nationally representative recent EDHS 2016. Ethiopia is the second-most populous Africa country, with nine regional states and two administrative cities. The EDHS 2016 used a stratified two-stage cluster sampling technique at which each regional state was stratified into urban and rural areas, giving 21 strata. In the first stage, 645 Enumeration Area (EA)/ clusters, 202 urban and 443 rural were selected proportional to EA size. In the second stage, 28 households per cluster were randomly selected from the newly created household listing. A detailed description of EDHS 2016 is provided in the full report of the survey [10].

Study population

The study population are postpartum women who either visited a health facility or were visited by a field worker at the community setting during the 12 months preceding the survey. Women were excluded if they were pregnant again within 12 months after the most recent birth (n=48) and if they had no contact with the health services delivery points during the previous 12 months (i.e. neither visited a health facility nor were visited by a field worker at the community setting) (n=840). Thus, the included study population was 1,526 women, which corresponded to a weighted sample size of 1,650 women (**Figure 1**).

Variables

- ### Covariates

After reviewing previous literature [12], we included sociodemographic characteristics, reproductive histories), and health service utilisation covariates for adjustment. The complete list of covariates used for adjustment and their definitions are included in the **supplementary material Table S1**.

For peer review only - http://bmjopen.bmj.com/site/about/guidelines.xhtml

- Exposure variable

The main exposure variable for this study was family planning counselling, which is defined as receiving family planning information either by a health service provider at a health facility or a family planning fieldworker within a community setting during the 12 months preceding the survey. We categorised the participants into two groups based on their exposure status. The first group consisted of participants who either visited a health facility or were visited by a family planning fieldworker in a community setting during the 12 months preceding the survey and received family planning information during either of the two contact points were labelled as '*counselled*' for family planning. The second group consisted of participants who either visited a health facility or were visited by a family planning fieldworker in a community setting during the 12 months preceding the survey but did not receive family planning information at either contact point were labelled as '*not counselled*' for family planning

- Outcome variable

The outcome of this study is postpartum modern contraceptive uptake, dichotomised to "yes" or "no". Modern contraceptive uptake was defined when participants were using sterilisation (female or male), condoms (female or male), injectables, oral pills, intrauterine contraceptive device (IUCD), implants, lactational amenorrhea (LAM) and standard calendar method at the time of the survey. Lack of uptake of a modern contraceptive method was defined as lack of use of any contraceptive method or traditional methods, such as abstaining and withdrawals [10].

Statistical analysis

We used a generalised linear regression model fitted with Poisson distribution and a log link function to examine the association between receipt of family planning counselling and postpartum modern contraceptive uptake after adjusting for sociodemographic characteristics, reproductive history, and health services utilisation. We applied two adjusted models. The first

For peer review only - http://bmjopen.bmj.com/site/about/guidelines.xhtml

model (*model 1*) adjusted for all covariates (**Table S1**). However, as the adjustment resulted in further loss of precision on the effect estimate, wide confidence intervals (CIs), we explored covariates that create a further loss of precision one by one and found to be a place of delivery, PNC visits, and postpartum period/ interview time. Later, we employed the second model (*model 2*), adjusting for all covariates, excluding a place of delivery, PNC, and postpartum period/interview time. We also run a sensitivity analysis to examine the association of family planning counselling and postpartum modern contraceptive uptake by stratifying the place of delivery, PNC visits and the postpartum period. The Chi-square test was used to check the distribution of covariates among counselled and non-counselled women. Multicollinearity among the covariate variables was checked using variance inflation factor (VIF). Unadjusted and adjusted relative risks (RRs) with 95% CIs were reported. All analyses accounted for the survey's non-response rate and complex sampling procedure and were undertaken with Stata 16 [24].

Patient and Public involvement

Patient or the public were not involved in this study

RESULTS

This study included 1,650 women who had contact with the health service during the 12 months preceding the EDHS survey. Less than half (48%) of the women reported that they received family planning counselling (**Table S2**). Of the women who received counselling, 655 (82%) and 435 (55%) were rural residents and had no education. Of the counselled women, 648 (82%) had attended at least one ANC visit during their last pregnancy. However, 401 (52%) and 561 (72%) were women who gave birth at home and did not attend PNC visits, respectively. Except for maternal age, parity, distance to a health facility, health insurance coverage and receipt of media-delivered family planning messages, all women's characteristics had similar distributions among those counselled and non-counselled. Of the counselled women, 361

For peer review only - http://bmjopen.bmj.com/site/about/guidelines.xhtml

(45%) were 30 years and above, whereas of the non-counselled women, 269 (21%) were 30 years and above. In reverse to this, of the counselled and non-counselled women, 231 (29%) and 318 (37%) were less than 25 years, respectively. Of those who received counselling, 289 (36%) received family planning messages from the media, while of those who did not receive counselling, only 208 (24%) received family planning messages from the media. Of the study women <6 months postpartum, 661 (84%) were postpartum amenorrhoeic, but only 473 (60%) met the LAM criteria. Indeed, more than three-quarters of women < 6 months postpartum in our study were not using modern contraceptive methods (**Table 1**).

Table 1. Characteristics of the study participants by the status of family planning counselling (n=1,650)

**Variable**	**Category**	**Family planning counselling**	
		**Not counselled (%)**<br>**N=856 (51.9)**	**Counselled (%)**<br>**N= 794 (48.1)**
Household wealth index	Lowest	162 (18.9)	155 (19.6)
	Middle	379 (44.3)	306 (38.5)
	Highest	315 (36.8)	333 (41.9)
Residence	Urban	116 (13.5)	139 (17.5)
	Rural	740 (86.5)	655 (82.5)
Region	Tigray	67 (7.8)	93 (11.8)
	Amhara	189 (22.1)	174 (22.0)
	Oromia	344 (34.4)	299 (37.6)
	SNNP	165 (19.3)	175 (22.0)
	Addis Ababa	30 (3.5)	25 (3.2)
	Others	61 (7.2)	27 (3.5)
Women's age	<25 years	318 (37.2)	231 (29.1)

For peer review only - http://bmjopen.bmj.com/site/about/guidelines.xhtml

	25-29 years	269 (31.4)	202 (25.4)
	≥30 years	269 (21.4)	361 (45.5)
Women's educational level	No education	447 (52.2)	435 (54.8)
	Primary	314 (36.7)	271 (34.2)
	Secondary and above	95 (11.1)	87 (11.0)
Women's current job	Not working	542 (63.3)	454 (57.2)
	Working	314 (36.7)	340 (42.8)
Religion	Christian	477 (55.7)	505 (63.6)
	Muslim	362 (42.3)	273 (34.4)
	Others	17 (2.0)	16 (2.0)
Women's decision-making autonomy	No autonomy	308 (39.0)	262 (34.1)
	Having autonomy	481 (61.0)	506 (65.9)
Parity	Primiparity	240 (28.5)	165 (20.8)
	Multiparity	431 (50.4)	419 (52.8)
	Grand multiparity	181 (21.1)	210 (26.4)
Wantdeness of the last pregnancy	Unintended	232 (27.1)	217 (27.3)
	Intended	624 (72.9)	577 (72.7)
Sex of the last child	Male	419 (49.0)	402 (50.7)
	Female	437 (51.0)	391 (49.3)
Survival status of the last child	Died	20 (2.3)	21 (2.7)
	Alive	836 (97.7)	772 (97.3)
ANC visits for the last pregnancy	No	200 (23.4)	146 (18.4)
	Yes	654 (76.6)	648 (81.6)
Place of delivery for the last	Home	472 (56.2)	401 (51.6)

For peer review only - http://bmjopen.bmj.com/site/about/guidelines.xhtml

birth	Health facility	368 (43.8)	376 (48.4)
PNC visits following the last birth	No	642 (75.9)	561 (71.9)
	Yes	180 (24.1)	199 (28.1)
Interview time/ postpartum period	0-6 moths	468 (54.7)	466 (58.7)
	7-12 months	388 (45.3)	328 (41.3)
Fertility intention	No more	288 (33.8)	340 (43.0)
	Have another	565 (66.2)	451 (57.0)
Receiving family planning messages from media	No	648 (75.6)	505 (63.6)
	Yes	208 (24.4)	289 (36.4)
Have health insurance	No	834 (97.4)	737 (92.9)
	Yes	22 (2.6)	56 (7.1)
Distance to a health facility	A big problem	501 (58.6)	409 (51.6)
	Not a big problem	355 (41.4)	385 (48.4)

For peer review only

There were 445 (27%) participants who used modern contraceptive methods at the survey time. The most used modern contraceptive method was injectable (18.4%%), followed by implants (4.7%).

Before adjustment, family planning counselling was associated with 27% higher postpartum modern contraceptive uptake (RR 1.27, 95% CI 1.00, 1.62). Moreover, the adjusted model (*model 1*) indicated that the risk of postpartum modern contraceptive uptake was 25% higher among women who have been counselled for family planning than those who did not receive counselling, although point estimates were imprecise (RR 1.32; 95% CI 1.04, 1.67). It was observed that place of delivery, PNC visits, and the postpartum period were the covariates that resulted in further loss of precision. The result of the adjusted model (*model 2*), excluding place of delivery, PNC visits, and postpartum period/interview time, also showed an association

For peer review only - http://bmjopen.bmj.com/site/about/guidelines.xhtml

between family planning counselling and postpartum modern contraceptive uptake (RR 1.25; 95% CI 0.99, 1.60) (**Table 2**).

Table 2. Association between family planning counselling and postpartum modern contraceptive uptake stratified by place of delivery, PNC visits and interview time postpartum

	**Unadjusted**	**Adjusted**[1]
All women	1.27 (1.00, 1.62)	1.25 (0.99, 1.60)
	Place of delivery	
Home delivery	1.35 (0.86, 2.13)	1.33 (0.83, 2.12)
Facility delivery	1.18 (0.90, 1.54)	1.27 (0.96, 1.67)
	PNC visits	
Had PNC visits	1.05 (0.73, 1.51)	1.25 (0.85, 1.83)
Had no PNC visits	1.39 (1.02, 1.90)	1.27 (0.92, 1.74)
	Interview time postpartum	
0-6 months postpartum	1.00 (0.66, 1.52)	0.90 (0.27, 2.96)
7-12 months postpartum	1.54 (1.17, 2.03)	1.60 (1.23, 2.07)

We performed a sensitivity analysis to further look at the effect of family planning counselling on postpartum contraceptive uptake by stratifying the place of delivery by home vs facility birth status, PNC visits as yes vs no, and the postpartum period into 0-6 vs 7-12 months. Although the effect estimates were imprecise (wide CIs), point estimates indicated that family

[1]Adjusted for household wealth status, residence, maternal age, maternal education, maternal employment, women's decision-making autonomy, religion, region, parity, survival status of the last-child, sex of last-child, wantdeness of last pregnancy, fertility intention, ANC , distance to health facility, health insurance coverage and receving of family planning messages from media

For peer review only - http://bmjopen.bmj.com/site/about/guidelines.xhtml

planning counselling improved contraceptive uptake regardless of the place of delivery and PNC visits. However, the group for which counselling was observed to be associated with improved contraceptive uptake gave birth 7-12 months prior to undertaking the survey.

DISCUSSION

This study evaluated the association between counselling for family planning and postpartum modern contraceptive uptake in Ethiopia using the EDHS 2016.

Our finding revealed that family planning counselling during the 12 months preceding the survey was associated with postpartum modern contraceptive uptake, which is supported by previous studies [2 20 21]. This was inconsistent with some previous studies from Ethiopia, which indicated a lack of association between family planning counselling and postpartum modern contraceptive uptake [22 25 26]. One reason for the inconsistency might be due to the differences quality of the family planning counselling. Although the previous studies conducted in Ethiopia on family planning counselling and postpartum contraceptive uptake did not describe the quality of the counselling, the quality can create a difference in adopting the contraceptive methods [27 28]. For example, a study conducted in Uttar Pradesh, India, showed that receiving higher quality family planning counselling improves the use of modern contraceptive methods [27]. Sociocultural differences might also explain the effect of family planning counselling on postpartum modern contraceptive uptake inconsistencies, as Ethiopia is multicultural with varied religious beliefs that may contribute to differences in perceptions and beliefs regarding contraceptive methods [28 29]. Therefore, even if women obtained counselling on family planning, this may not necessarily be translated to contraceptive use. Notable examples include sociocultural influences, such as gender inequality of power in decision-making or religious prohibitions to use contraceptive methods [28 30].

Our study also indicated that only half of the women received family planning counselling (48%), which is slightly higher than the previous studies in Ethiopia, which indicated that only

For peer review only - http://bmjopen.bmj.com/site/about/guidelines.xhtml

28- 35% of women were provided family planning counselling [31 32]. This discrepancy might be due to the difference in family planning counselling definition. Unlike the previous studies that used only facility-based counselling, our definition includes counselling provided in facility and community settings. Nonetheless, the result indicates that despite the fact that family planning services are integrated with maternal and child health services at the policy level [17 29], there remains a gap in the universal adoption of the recommendation by healthcare providers to counsel family planning, resulting in a higher proportion of women who missed the opportunity to be counselled. Although further research is needed on the barriers to the missed opportunity of providing family counselling while visiting service delivery points, previous qualitative research indicated that lack of awareness by the service providers of the national guidelines and the lack of the guidelines in the health facilities were the main barrier to not adhering on it [33]. The structural barriers, such as shortage of infrastructure and equipment and the unbalanced client-to-provider ratios within the Ethiopian health care system [34], might contribute to the high missed opportunity to provide family planning counselling.

Adjusted point estimates differed after additional adjustment for the place of delivery, PNC, and the postpartum period/interview time, implying that the pathway through which family planning counselling affects contraceptive uptake may act through these variables. We conducted additional sensitivity analyses to investigate further these pathways, which indicated that family planning counselling improved postpartum contraceptive uptake regardless of the place of delivery and PNC visits. However, we also observed that counselling was associated with improved postpartum contraceptive uptake only for women interviewed at least seven after giving birth. Delivery at a health facility or receipt of PNC that includes family planning counselling may not translate an increased likelihood of modern contraceptive uptake if women believe that they would be free of the risk of pregnancy while amenorrhoeic following delivery. Ethiopian women may consider resumption menses as critical timing for starting contraception

For peer review only - http://bmjopen.bmj.com/site/about/guidelines.xhtml

in the postpartum period [26]. Ethiopian women may also believe that they are not at risk of pregnancy in the first six months after giving birth if they are breastfeeding their baby, whether or not breastfeeding is exclusive [35]. The findings of stronger associations for women interviewed at least seven months after giving birth could also be because there might be a greater proportion of women who have re-engaged in sexual activity during this period than in the first six months.

Overall, our findings highlighted the importance of integration of family planning counselling with other health services, particularly with maternal and child health services. This helps dispel misperceptions and enable women to make informed decisions that would significantly reduce missed opportunities, thereby improving family planning counselling coverage and the uptake of postpartum modern contraception. Hence, the Ethiopian government and partner organisations with the remit of family planning, maternal and child health should work to translate the plans documented in the National Family Planning Guideline and the National Reproductive Health Strategy.

This is the first study that assessed the association between family planning counselling and postpartum modern contraceptive uptake at the national level and provides insight for government and partner organisations on the gaps in the provision of family planning counselling. However, this study has several limitations that need to be considered when interpreting our findings. The type and frequency of family planning information and the type of health care provider who provided the counselling were not collected within the EDHS. Consequently, we were unable to examine the quality of counselling provided. Meanwhile, as the women were asked to provide family planning information for the 12 months prior to undertaking the survey, information before this time might have contributed to the association between family planning counselling and postpartum modern contraceptive uptake. Furthermore, although the data was based on self-reported information that occurred in the

For peer review only - http://bmjopen.bmj.com/site/about/guidelines.xhtml

preceding 12 months, we cannot completely rule out the influence of recall bias. Lastly, due to the nature of the cross-sectional study design, it is not easy to determine the causal relationship between family planning counselling and postpartum modern contraceptive uptake.

CONCLUSION

Significant numbers of postpartum women did not receive family planning counselling despite contact with health service contact points during the preceding 12 months. We found that postpartum modern contraceptive uptake was low. Nonetheless, our findings reveal that family planning counselling is associated with improving postpartum modern contraceptive uptake in Ethiopia. Hence, besides preparing guidelines and working documents, the government should work to fully implement the integration of family planning counselling at all levels of service delivery points. Additionally, all service providers should deliver family planning counselling by complying with the national guideline, and postpartum counselling should stress the early initiation of modern contraceptives after giving birth. On top of these, continuous monitoring and evaluation of family planning counselling integration would help to reduce the missed opportunities and improve contraceptive uptake.

Author Contributions

Kalayu Brhane Mruts: Conceptualisation, Formal analysis, Data Curation, Writing- Original Draft, Writing-Review & Editing, Visualisation. **Gizachew A Tessema:** Writing-Review & Editing, Visualisation. **Jennifer Dunne**: Writing-Review & Editing, Visualisation. **Amanuel Tesfay Gebremedhin**: Writing-Review & Editing, Visualisation. **Jane A Scott**: Writing-Review & Editing, Visualisation. **Gavin Pereira**: Writing-Review & Editing, Visualisation

Ethical statements

Ethical approval

As this study used publicly available secondary data, ethical approval was not required.

Patient consent for publication: not required

For peer review only - http://bmjopen.bmj.com/site/about/guidelines.xhtml

Data availability

Data are available in public open access in the DHS program repository (https://dhsprogram.com/.). Registration is required to access the dataset.

Acknowledgments

We are grateful to the DHS program for providing access to the Ethiopian Demographic and Health Survey data

Declaration of conflicting interests

The authors have declared that no competing interests exist

Source of Funding

This research was supported by funding by the National Health and Medical Research Council Project Grants #1099655 and #1173991 for GP, and #1195716 for GAT and institutional funding for the WA Health and Artificial Intelligence Consortium, and the Research Council of Norway through its Centres of Excellence funding scheme #262700 for GP. In addition, KBM was supported by an Australian Government Research Training Program International Scholarship. However, the funders had no role in study design, data collection, analysis, interpretation of the results and decided to publish or prepare the manuscript.

REFERENCES

1. Cleland J, Conde-Agudelo A, Peterson H, et al. Contraception and health. *Lancet* 2012;380(9837):149-56. doi: 10.1016/S0140-6736(12)60609-6 [published Online First: 2012/07/13]
2. Amour C, Manongi RN, Mahande MJ, et al. Missed opportunity for family planning counselling along the continuum of care in Arusha region, Tanzania. *PLOS ONE* 2021;16(7):e0250988. doi: 10.1371/journal.pone.0250988
3. Moore Z, Pfitzer A, Gubin R, et al. Missed opportunities for family planning: an analysis of pregnancy risk and contraceptive method use among postpartum women in 21 low- and middle-income countries. *Contraception* 2015;92(1):31-39. doi: 10.1016/j.contraception.2015.03.007
4. Ameyaw EK, Budu E, Sambah F, et al. Prevalence and determinants of unintended pregnancy in sub-Saharan Africa: A multi-country analysis of demographic and health surveys. *PloS one* 2019;14(8):e0220970-e70. doi: 10.1371/journal.pone.0220970

For peer review only - http://bmjopen.bmj.com/site/about/guidelines.xhtml

5. United Nations Interagency Group for Child Mortality Estimation. Levels & Trends in Child Mortality: Report 2020: Estimates Developed by the UN Inter-Agency Group for Child Mortality Estimation: United Nations Children's Fund, 2020.
6. World Health Organization. Trends in maternal mortality 2000 to 2017: estimates by WHO, UNICEF, UNFPA, World Bank Group and the United Nations Population Division, 2019.
7. Mahande MJ, Obure J. Effect of interpregnancy interval on adverse pregnancy outcomes in northern Tanzania: a registry-based retrospective cohort study. *BMC Pregnancy Childbirth* 2016;16(1):140. doi: 10.1186/s12884-016-0929-5 [published Online First: 2016/06/09]
8. Rutstein SO. Effects of preceding birth intervals on neonatal, infant and under-five years mortality and nutritional status in developing countries: evidence from the demographic and health surveys. *International Journal of Gynecology & Obstetrics* 2005;89:S7-S24. doi: 10.1016/j.ijgo.2004.11.012
9. Tesema GA, Tessema ZT, Tamirat KS, et al. Prevalence of stillbirth and its associated factors in East Africa: generalised linear mixed modeling. *BMC Pregnancy Childbirth* 2021;21(1):414. doi: 10.1186/s12884-021-03883-6 [published Online First: 2021/06/04]
10. Central Statistical Agency - CSA/Ethiopia, ICF. Ethiopia Demographic and Health Survey 2016. Addis Ababa, Ethiopia: CSA and ICF, 2017.
11. Teshale AB, Tesema GA. Magnitude and associated factors of unintended pregnancy in Ethiopia: a multilevel analysis using 2016 EDHS data. *BMC Pregnancy and Childbirth* 2020;20(1) doi: 10.1186/s12884-020-03024-5
12. Dagnew GW, Asresie MB, Fekadu GA, et al. Modern contraceptive use and factors associated with use among postpartum women in Ethiopia; further analysis of the 2016 Ethiopia demographic and health survey data. *BMC Public Health* 2020;20(1):661. doi: 10.1186/s12889-020-08802-6
13. Endalamaw A, Engeda EH, Ekubagewargies DT, et al. Low birth weight and its associated factors in Ethiopia: a systematic review and meta-analysis. *Ital J Pediatr* 2018;44(1):141. doi: 10.1186/s13052-018-0586-6 [published Online First: 2018/11/28]
14. Jena BH, Biks GA, Gelaye KA, et al. Magnitude and trend of perinatal mortality and its relationship with inter-pregnancy interval in Ethiopia: a systematic review and meta-analysis. *BMC Pregnancy and Childbirth* 2020;20(1) doi: 10.1186/s12884-020-03089-2
15. Muchie KF, Lakew AM, Teshome DF, et al. Epidemiology of preterm birth in Ethiopia: systematic review and meta-analysis. *BMC Pregnancy Childbirth* 2020;20(1):574. doi: 10.1186/s12884-020-03271-6 [published Online First: 2020/10/01]
16. World Health Organization. Programming strategies for postpartum family planning, 2013.
17. Federal Democratic Republic of Ethiopia MoH. National Guideline for Family Planning Services in Ethiopia, 2020.
18. Dehlendorf C, Krajewski C, Borrero S. Contraceptive counseling: best practices to ensure quality communication and enable effective contraceptive use. *Clinical obstetrics and gynecology* 2014;57(4):659.
19. Zapata LB, Murtaza S, Whiteman MK, et al. Contraceptive counseling and postpartum contraceptive use. *American journal of obstetrics and gynecology* 2015;212(2):171. e1-71. e8.
20. Tafa L, Worku Y. Family planning utilisation and associated factors among postpartum women in Addis Ababa, Ethiopia, 2018. *PLoS One* 2021;16(1):e0245123. doi: 10.1371/journal.pone.0245123 [published Online First: 2021/01/23]
21. Tafere TE, Afework MF, Yalew AW. Counseling on family planning during ANC service increases the likelihood of postpartum family planning use in Bahir Dar City Administration, Northwest Ethiopia: a prospective follow up study. *Contracept Reprod Med* 2018;3:28. doi: 10.1186/s40834-018-0081-x [published Online First: 2019/01/05]
22. Woldu BF, Ermolo TL, Lemu LG, et al. Long-acting reversible contraception utilisation and associated factors among women in extended postpartum period in Hossana town, southern

For peer review only - http://bmjopen.bmj.com/site/about/guidelines.xhtml

Ethiopia: cross sectional study. *Contracept Reprod Med* 2020;5(1):10. doi: 10.1186/s40834-020-00117-6 [published Online First: 2020/08/11]

23. Zimmerman LA, Yi Y, Yihdego M, et al. Effect of integrating maternal health services and family planning services on postpartum family planning behavior in Ethiopia: results from a longitudinal survey. *BMC Public Health* 2019;19(1):1448. doi: 10.1186/s12889-019-7703-3 [published Online First: 2019/11/07]
24. StataCorp L. Stata Statistical Software. Release 16.[software]. College Station, TX: Stata Press. Available at: https://www. stata. com/. Accessed September, 2019.
25. Gebremariam A, Gebremariam H. Contraceptive use among lactating women in Ganta-Afeshum District, Eastern Tigray, Northern Ethiopia, 2015: a cross sectional study. *BMC Pregnancy Childbirth* 2017;17(1):421. doi: 10.1186/s12884-017-1613-0 [published Online First: 2017/12/17]
26. Gejo NG, Anshebo AA, Dinsa LH. Postpartum modern contraceptive use and associated factors in Hossana town. *PLoS One* 2019;14(5):e0217167. doi: 10.1371/journal.pone.0217167 [published Online First: 2019/05/23]
27. Dehingia N, Dixit A, Averbach S, et al. Family planning counseling and its associations with modern contraceptive use, initiation, and continuation in rural Uttar Pradesh, India. *Reprod Health* 2019;16(1):178. doi: 10.1186/s12978-019-0844-0 [published Online First: 2019/12/14]
28. Edberg M, Sedlander E, Rimal R, et al. Planned social network change and modern contraceptive use in a rural Ethiopian community. *Journal of Global Health Reports* 2018;2 doi: 10.29392/joghr.2.e2018034
29. Ethiopia Federal Ministry of Health. National Reproductive Health Strategy 2016-2020: Federal Ministry of Health (FMOH) Addis Ababa, Ethiopia, 2016.
30. Tigabu S, Demelew T, Seid A, et al. Socioeconomic and religious differentials in contraceptive uptake in western Ethiopia: a mixed-methods phenomenological study. *BMC Women's Health* 2018;18(1) doi: 10.1186/s12905-018-0580-6
31. Teshome A, Birara M, Rominski SD. Quality of family planning counseling among women attending prenatal care at a hospital in Addis Ababa, Ethiopia. *International Journal of Gynecology and Obstetrics* 2017;137(2):174-79. doi: 10.1002/ijgo.12110
32. Abdulreshid M, Dadi HB. Assessment of Family Planning Counseling Provided for Postpartum Women and Associated Factors. *International Journal of Reproductive Medicine* 2020;2020:2649340. doi: 10.1155/2020/2649340
33. Tessema G. A. Health provider perspectives on implementation of family planning guidelines in family planning services. The University of Adelaide, 2018.
34. Assefa Y, Gelaw YA, Hill PS, et al. Community health extension program of Ethiopia, 2003-2018: successes and challenges toward universal coverage for primary healthcare services. *Global Health* 2019;15(1):24. doi: 10.1186/s12992-019-0470-1 [published Online First: 2019/03/28]
35. Labbok MH, Hight-Laukaran V, Peterson AE, et al. Multicenter study of the Lactational Amenorrhea Method (LAM): I. Efficacy, duration, and implications for clinical application. *Contraception* 1997;55(6):327-36. doi: 10.1016/s0010-7824(97)00040-1 [published Online First: 1997/06/01]

Figure 1. Participant's selection

Note: The sample weights account for the probability of selection and interview in the EDHS.

For peer review only - http://bmjopen.bmj.com/site/about/guidelines.xhtml

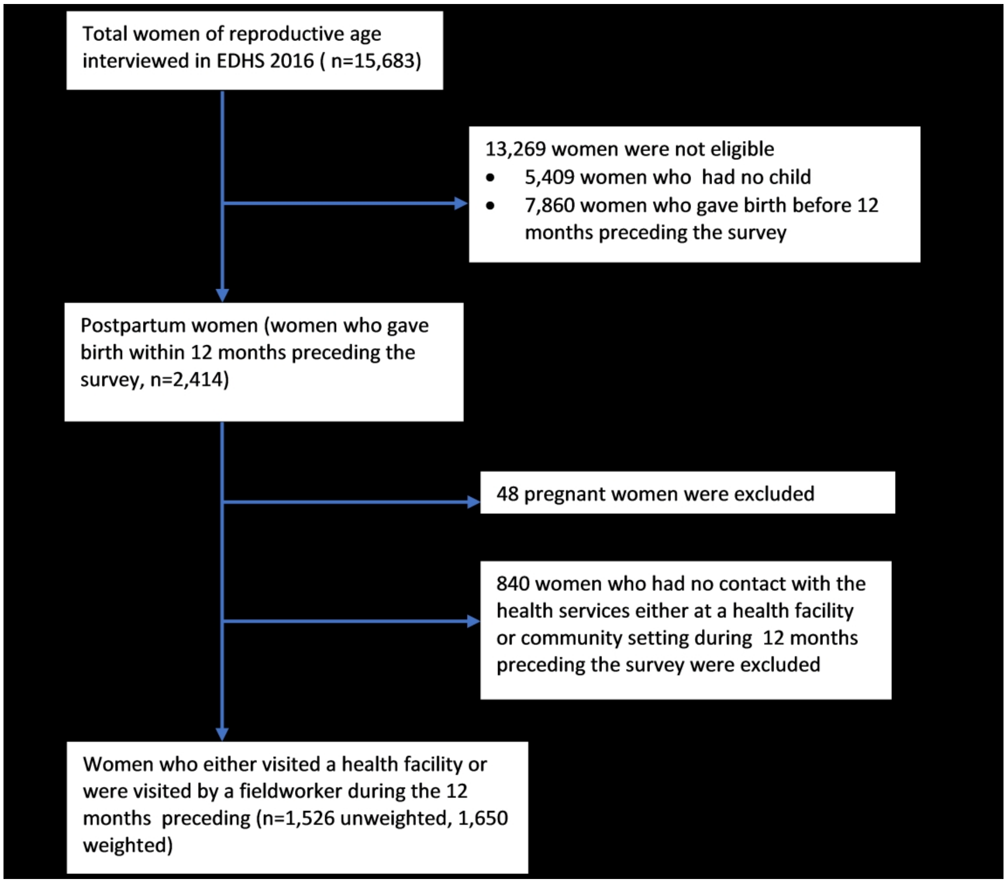

Figure 1. Participant's selection

665x581mm (144 x 144 DPI)

Table S1; List of covariates and their definition

Variable	Definition
Household wealth quintile	The household wealth index was computed by principal component analysis (PCA) using the household's ownership of selected items, such as radio and television, and possession of household animals, including oxen, cow, camel; and materials used for housing construction, and types of water access and sanitation facilities taking into consideration of urban-rural difference. We categorised the wealth index score into three equal parts labelled as lowest, middle, and highest
Residence	Participant place of residence coded as urban and rural. Urban was defined as a locality with 2,000 or more inhabitants. Moreover, all administrative capitals, including Region, Zone and Woreda, and localities in which urban dwellers' associations were established were considered urban residences, irrespective of the population size. Rural residences comprise all areas not classified as urban.
Region	Ethiopia has nine regional states and two city administrations. For this study purpose, we merged the emerging regional states of Ethiopia, such as Afar, Somali, Benishangul-Gumuz, Harari and Dire Dawa, into one group as others. Hence, the region categorised into Tigray, Amhara, Oromia, SNNP, Addis Ababa and others
Women's age	Women's age was grouped into <25 years, 25-29 years, and ≥30 years
Women's educational status	Women's education status is available in EDHS coded as no education, primary, secondary, and higher. Since the frequency of women who had a higher level of education was low, we merged the secondary and higher into 'secondary and above.'
Women's employment	Participants were grouped as employed when they reported as working within seven days preceding the survey, including women who did not work in the past seven days but who are regularly employed and were absent from work for leave, illness, vacation, or any other such reason, otherwise as not employed
Marital status	The marital status was categorised as married/in union, including married and living with a partner, and not married/in union, including never in union, widowed, divorced, and no longer living together/separated

For peer review only

Women's religion	Women's religion is available in the EDHS coded as Orthodox, Muslim, Protestant, Catholic, Traditional, and other. We merged Orthodox, Protestant and Catholic into Christian and Traditional and others into other. Hence, religion in this study was categorised as Christian, Muslim and Others
Parity	Parity of the participant women was categorised into primiparity (para 1), multiparity (para 2-4) and grand multiparity (para ≥5)
Wantdeness of the last child	Wantdeness of the last child grouped into planned, unplanned, and unwanted
Sex of last-child	Sex of the last child was categorised as male or female
Survival status of the last child	Survival status of the last child categorised as dead and alive
ANC for the pregnancy of the last birth	ANC visit at least once during the pregnancy of last birth was coded as yes, if not as no
Place of delivery for the last birth	Place of delivery was labelled as home if delivered at their home or any other home, and health facility if delivered in any one of the health facilities- public, private, or non-governmental health facility.
PNC following the last birth	PNC was categorised as yes when they received any checking their health status either at a health facility or at home within six weeks following the last birth; otherwise, no
Postpartum period/interview time	The postpartum period is the time between the birth of the last child and the interview date and was categorised into 0-6 months and 7-12 months
Future fertility intention	Future fertility intention was categorised as have more, no more and undecided
Receiving family planning messages from media	receiving of family planning messages from media was labelled as 'yes' when women received any family planning messages from any one of the following: radio, television, newspaper/magazine or text message on mobile phone in the past few months preceding the survey, and 'no' if did not receive any message from any one of the media channels.
Distance to a health facility for getting medical help	The distance to a health facility for getting medical help was captured by a self-reported response as a big problem vs not a big problem
Health insurance	Health insurance was categorised as yes and no, corresponding for having and not having health insurance, respectively.

For peer review only

Table S2; Characteristics of the study participants (n=1,650)

Variable	Category	Frequency	Percentage
Household wealth index	Lowest	317	19.2
	Middle	685	41.5
	Highest	647	39.2
Residence	Urban	255	15.4
	Rural	1,395	84.6
Region	Tigray	160	9.7
	Amhara	363	22.0
	Oromia	642	38.9
	SNNP	340	20.6
	Addis Ababa	55	3.3
	Others	89	5.4
Women's age at birth of the last child	<25 years	634	38.4
	25-29 years	456	27.7
	≥30 years	559	33.9
Women's educational level	No education	881	53.4
	Primary	586	35.5
	Secondary and above	183	11.1
Marital status	Not married/in union	73	4.4
	Married/in union	1,577	95.6
Women's current job	Not working	996	60.4
	Working	654	39.6
Religion	Christian	981	59.5
	Muslim	635	38.5
	Others	33	2.0
Women's decision-making autonomy	No autonomy	570	36.6
	Having autonomy	987	63.4
Parity	Primiparity	409	24.8
	Multiparity	850	51.5
	Grand multiparity	390	23.7
Wantdeness of the last pregnancy	Unintended	449	27.2
	Intended	1,200	72.8
Sex of the last child	Male	822	49.8
	Female	828	50.2
Survival status of the last child	Died	41	2.5
	Alive	1,608	97.5
ANC visits for pregnancy of the last birth	No	346	21.0
	Yes	1,302	79.0
Place of delivery for the last birth	Home	873	54.0
	Health facility	744	46.0
PNC visits following the last birth	No	1,203	76.1
	Yes	379	23.9
Postpartum period/ Interview time	0-6 moths	934	56.6
	7-12 months	716	43.4
Fertility intention	No more	629	38.2
	Have another	1,017	61.8
receiving of family planning messages from media	No	1,152	69.8
	Yes	498	30.2
Distance to a health facility	A big problem	911	55.2
	Not a big problem	739	44.8
Have health insurance	No	1,571	95.2
	Yes	78	4.8
Family planning counselling	Not counselled	856	51.9
	Counselled	794	48.1

For peer review only

Table S3; The sample size for the adjusted and unadjusted models among all and specific women

Samples		**Unadjusted**	**Adjusted**
All women		1,650	1,550
Place of delivery	Home delivery	873	831
	Facility delivery	743	689
PNC visits	Had PNC visits	423	394
	Had no PNC visits	1,203	1,134
Interview time postpartum	0-6 months postpartum	934	888
	7-12 months postpartum	716	662

For peer review only

Reporting checklist for cross sectional study.

Based on the STROBE cross sectional guidelines.

Instructions to authors

Complete this checklist by entering the page numbers from your manuscript where readers will find each of the items listed below.

Your article may not currently address all the items on the checklist. Please modify your text to include the missing information. If you are certain that an item does not apply, please write "n/a" and provide a short explanation.

Upload your completed checklist as an extra file when you submit to a journal.

In your methods section, say that you used the STROBE cross sectionalreporting guidelines, and cite them as:

von Elm E, Altman DG, Egger M, Pocock SJ, Gotzsche PC, Vandenbroucke JP. The Strengthening the Reporting of Observational Studies in Epidemiology (STROBE) Statement: guidelines for reporting observational studies.

		Reporting Item	Page Number
**Title and abstract**			
Title	#1a	Indicate the study’s design with a commonly used term in the title or the abstract	1

For peer review only

Abstract	#1b	Provide in the abstract an informative and balanced summary of what was done and what was found	2
**Introduction**			
Background / rationale	#2	Explain the scientific background and rationale for the investigation being reported	4-5
Objectives	#3	State specific objectives, including any prespecified hypotheses	5
**Methods**			
Study design	#4	Present key elements of study design early in the paper	6
Setting	#5	Describe the setting, locations, and relevant dates, including periods of recruitment, exposure, follow-up, and data collection	6
Eligibility criteria	#6a	Give the eligibility criteria, and the sources and methods of selection of participants.	6
	#7	Clearly define all outcomes, exposures, predictors, potential confounders, and effect modifiers. Give diagnostic criteria, if applicable	6-7
Data sources / measurement	#8	For each variable of interest give sources of data and details of methods of assessment (measurement). Describe comparability of assessment methods if there is more than one group. Give information separately for for exposed and unexposed groups if applicable.	7

For peer review only

Bias	#9	Describe any efforts to address potential sources of bias	n/a
Study size	#10	Explain how the study size was arrived at	6
Quantitative variables	#11	Explain how quantitative variables were handled in the analyses. If applicable, describe which groupings were chosen, and why	7-8
Statistical methods	#12a	Describe all statistical methods, including those used to control for confounding	7-8
Statistical methods	#12b	Describe any methods used to examine subgroups and interactions	8
Statistical methods	#12c	Explain how missing data were addressed	n/a
Statistical methods	#12d	If applicable, describe analytical methods taking account of sampling strategy	8
Statistical methods	#12e	Describe any sensitivity analyses	8
**Results**			
Participants	#13a	Report numbers of individuals at each stage of study—eg numbers potentially eligible, examined for eligibility, confirmed eligible, included in the study, completing follow-up, and analysed. Give information separately for for exposed and unexposed groups if applicable.	8
Participants	#13b	Give reasons for non-participation at each stage	6

For peer review only

Participants	#13c	Consider use of a flow diagram	6
Descriptive data	#14a	Give characteristics of study participants (eg demographic, clinical, social) and information on exposures and potential confounders. Give information separately for exposed and unexposed groups if applicable.	8
Descriptive data	#14b	Indicate number of participants with missing data for each variable of interest	8
Outcome data	#15	Report numbers of outcome events or summary measures. Give information separately for exposed and unexposed groups if applicable.	8-11
Main results	#16a	Give unadjusted estimates and, if applicable, confounder-adjusted estimates and their precision (eg, 95% confidence interval). Make clear which confounders were adjusted for and why they were included	11-12
Main results	#16b	Report category boundaries when continuous variables were categorized	n/a
Main results	#16c	If relevant, consider translating estimates of relative risk into absolute risk for a meaningful time period	n/a
Other analyses	#17	Report other analyses done—e.g., analyses of subgroups and interactions, and sensitivity analyses	12-13
**Discussion**			
Key results	#18	Summarise key results with reference to study objectives	13

For peer review only

Limitations	#19	Discuss limitations of the study, taking into account sources of potential bias or imprecision. Discuss both direction and magnitude of any potential bias.	15
Interpretation	#20	Give a cautious overall interpretation considering objectives, limitations, multiplicity of analyses, results from similar studies, and other relevant evidence.	13-15
Generalisability	#21	Discuss the generalisability (external validity) of the study results	15
**Other Information**			
Funding	#22	Give the source of funding and the role of the funders for the present study and, if applicable, for the original study on which the present article is based	17

The STROBE checklist is distributed under the terms of the Creative Commons Attribution License CC-BY. This checklist was completed on 15. December 2021 using https://www.goodreports.org/, a tool made by the EQUATOR Network in collaboration with Penelope.ai

For peer review only

BMJ Open

Does family planning counselling during health service contact improve postpartum modern contraceptive uptake in Ethiopia? A nationwide cross-sectional study

Journal:	*BMJ Open*
Manuscript ID	bmjopen-2021-060308.R1
Article Type:	Original research
Date Submitted by the Author:	25-Mar-2022
Complete List of Authors:	Mruts, Kalayu Brhane; Curtin University, School of Population Health; Debre Berhan University, School of Public Health<br>Tessema, Gizachew; Curtin University, School of Population Health ; The University of Adelaide, School of Public Health<br>Dunne, Jennifer; Curtin University, School of Population Health<br>Gebremedhin, Amanuel Tesfay; Curtin University, School of Population Health; Wesfarmers Centre for Vaccine and Infectious Disease, Telethon Kids Institute, University of Western Australia, Perth, Australia<br>Scott, Jane; Curtin University, School of Population Health<br>Pereira, G.F; Curtin University, School of Population Health; Centre for Fertility and Health (CeFH), Norwegian Institute of Public Health, School of Population
**Primary Subject Heading**:	Public health
Secondary Subject Heading:	Public health, Reproductive medicine, Epidemiology, Global health
Keywords:	PUBLIC HEALTH, EPIDEMIOLOGY, Reproductive medicine < GYNAECOLOGY

SCHOLARONE™
Manuscripts

I, the Submitting Author has the right to grant and does grant on behalf of all authors of the Work (as defined in the below author licence), an exclusive licence and/or a non-exclusive licence for contributions from authors who are: i) UK Crown employees; ii) where BMJ has agreed a CC-BY licence shall apply, and/or iii) in accordance with the terms applicable for US Federal Government officers or employees acting as part of their official duties; on a worldwide, perpetual, irrevocable, royalty-free basis to BMJ Publishing Group Ltd ("BMJ") its licensees and where the relevant Journal is co-owned by BMJ to the co-owners of the Journal, to publish the Work in this journal and any other BMJ products and to exploit all rights, as set out in our licence.

The Submitting Author accepts and understands that any supply made under these terms is made by BMJ to the Submitting Author unless you are acting as an employee on behalf of your employer or a postgraduate student of an affiliated institution which is paying any applicable article publishing charge ("APC") for Open Access articles. Where the Submitting Author wishes to make the Work available on an Open Access basis (and intends to pay the relevant APC), the terms of reuse of such Open Access shall be governed by a Creative Commons licence – details of these licences and which Creative Commons licence will apply to this Work are set out in our licence referred to above.

Other than as permitted in any relevant BMJ Author's Self Archiving Policies, I confirm this Work has not been accepted for publication elsewhere, is not being considered for publication elsewhere and does not duplicate material already published. I confirm all authors consent to publication of this Work and authorise the granting of this licence.

For peer review only

Does family planning counselling during health service contact improve postpartum modern contraceptive uptake in Ethiopia? A nationwide cross-sectional study

Kalayu Brhane Mruts[1,2] *, Gizachew A Tessema[1,3], Jennifer Dunne[1], Amanuel Tesfay Gebremedhin[1,4], Jane A. Scott[1], Gavin Pereira[1,5,6]

[1] School of Population Health, Curtin University, Perth, Australia

[2] School of Public Health, College of Health Science, Debre Berhan University, Debre Berhan, Ethiopia

[3] School of Public Health, University of Adelaide, Adelaide, Australia

[4] Wesfarmers Centre for Vaccine and Infectious Disease, Telethon Kids Institute, University of Western Australia, Perth, Australia

[5] Centre for Fertility and Health (CeFH), Norwegian Institute of Public Health, Oslo, Norway

[6] enAble Institute, Curtin University, Perth Australia

* **Corresponding Author:**

Kalayu Brhane Mruts

Curtin School of Population Health, Curtin University

GPO Box U1987, Perth Western Australia 6845

Email: kalayu.brhanemruts@postgrad.curtin.edu.au

Phone: +61 478014036

World count: abstract=253; main text=2,912

For peer review only - http://bmjopen.bmj.com/site/about/guidelines.xhtml

ABSTRACT

Objective: This study examined the association between family planning counselling receipt during the 12 months preceding the survey and postpartum modern contraceptive uptake in Ethiopia. We hypothesised that women who had received family planning counselling either within the community setting by a field health worker or at a health facility by a healthcare attendant during the 12 months preceding the survey would adopt postpartum modern contraceptives.

Design: We used a cross-sectional study of the Ethiopian Demographic and Health survey conducted in 2016

Setting: Ethiopia

Participants: 1650 women who gave birth during the 12 months and had contact with service delivery points during the 12 months preceding the survey

Primary outcome: A weighted modified Poisson regression model estimated an adjusted relative risk (RR) of postpartum modern contraceptives.

Results: Approximately half (48%) of the women have missed the opportunity to receive family planning counselling at the health service contact points during the 12 months preceding the survey. The postpartum modern contraceptive uptake was 27%. Two hundred forty-two (30%) and 204 (24%) of the counselled and not-counselled women used postpartum modern contraceptive methods, respectively. Compared to women who did not receive counselling for family planning, women who received counselling had higher contraceptive uptake (RR 1.32, 95% CI 1.04, 1.67).

Conclusion: Significant numbers of women have missed the opportunity of receiving family planning counselling during contact with health service delivery points. Modern contraceptive uptake among postpartum women was low. Despite this, our findings revealed that family planning counselling was associated with improved postpartum modern contraceptive uptake.

For peer review only - http://bmjopen.bmj.com/site/about/guidelines.xhtml

Keywords: Contraceptive uptake, postpartum, family planning counselling, modern contraceptive, Ethiopia

For peer review only

For peer review only - http://bmjopen.bmj.com/site/about/guidelines.xhtml

Article Summary

Strengths and limitations of this study

- This is a first study that examined the association between family planning counselling and postpartum modern contraceptive uptake using large national survey data that enables our finding representative
- Adjustments were made for potential confounders
- The family planning counselling and contraceptive uptake were measured using self-reported with no available previous counselling information that might right in recall bias
- This is a cross-sectional that could not assure the causal relationship between family planning counselling and postpartum modern contraceptive uptake

For peer review only - http://bmjopen.bmj.com/site/about/guidelines.xhtml

INTRODUCTION

Delay in the initiation of modern contraceptives in the postpartum period is highly likely to result in unintended and closely spaced pregnancies [1]. Countries in sub-Saharan Africa (SSA) have the lowest postpartum modern contraceptive uptake (ref), accounting for the highest-burden of unintended and closely spaced pregnancies [2-4]. Unintended and closely spaced pregnancies are public health issues strongly associated with increased adverse health consequences for the mother and birth outcomes, such as preterm birth, stillbirth, low birth weight and neonatal and infant mortality [5-7].

Although modern contraceptive uptake has improved in Ethiopia over the last two decades [8], the postpartum modern contraceptive uptake is still low[9 10], resulting in high rates of unintended pregnancy and fertility rates [8 9 11]. For example, a study in Ethiopia using the Ethiopian Demographic and Health (EDHS) 2016 indicated that only 23% of women in the extended postpartum period- the first 12 months following the recent childbirth-using modern contraceptive [9]. Additionally, 27% of Ethiopian women conceive their pregnancy as unintended[11]. Moreover, it is reported that a short interbirth interval had been associated with stillbirth, preterm birth, neonatal mortality, and low birth weight in Ethiopia [12-14]. Provision of family planning counselling during pregnancy, childbirth and postpartum periods is one of the key strategies to improve postpartum modern contraceptive uptake and space subsequent pregnancies [15]. Furthermore, increasing pregnancy spacing could prevent up to 32% of maternal and 21% of child mortalities [1]. In order to improve postpartum contraceptive uptake in low and middle-income countries, the World Health Organisation (WHO) recommends that women receive family planning counselling integrated within maternal and child health services, such as antenatal care (ANC), childbirth, postnatal care (PNC), and well-baby care, immunisation and growth monitoring [16]. To increase access to contraception, the Ethiopian Ministry of Health also recommends that women receive family planning counselling at all the

For peer review only - http://bmjopen.bmj.com/site/about/guidelines.xhtml

health service contact points, such as health facilities and community settings [17]. The health facilities in the country (public, private, or non-governmental) are expected to provide family planning services integrated with all the health services [17]. Additionally, in the community settings, more than 39,000 health extension workers (HEWs) work across 16,000 villages, mainly in the rural community, to provide health education and counselling on maternal and child health and nutrition, including family planning counselling. Providing appropriate family planning counselling about the potential risks and side effects of modern contraceptive methods is essential for women to avoid misperceptions, increase knowledge, and make informed decisions [18 19].

Despite the strong WHO and Ethiopian Ministry of Health recommendations, Ethiopia lacks research at the national level assessing the association between family planning counselling and postpartum modern contraceptive uptake. Previous studies, conducted with small samples at the district level, have reported mixed evidence [20-23]. This study aims to assess the association between family planning counselling, provided either at the health facility by a health worker or at the community setting by a family planning field worker and postpartum modern contraceptive uptake in Ethiopia using the most recent national representative EDHS 2016. This study will increase our understanding of the contribution of the family planning counselling provided in Ethiopia on postpartum modern contraceptive uptake. The outcomes of this study will provide decision-makers and program managers with an evidence-based to assess and strengthen the counselling services of family planning. Additionally, it will motivate health workers to provide appropriate family planning information during the counselling sessions.

For peer review only - http://bmjopen.bmj.com/site/about/guidelines.xhtml

METHODS

Study design and setting

We used cross-sectional data from the nationally representative recent EDHS 2016. Ethiopia is the second-most populous Africa country, with 11 regional states and two administrative cities. The EDHS 2016 used a stratified two-stage cluster sampling technique at which each regional state was stratified into urban and rural areas, giving 21 strata. In the first stage, 645 Enumeration Area (EA)/ clusters, 202 urban and 443 rural were selected proportional to EA size. In the second stage, 28 households per cluster were randomly selected from the newly created household listing. A detailed description of EDHS 2016 is provided in the full report of the survey [8].

Study population

The study population are postpartum women who either visited a health facility or were visited by a field worker at the community setting during the 12 months preceding the survey. Women were excluded if they were pregnant again within 12 months after the most recent birth (n=48) and if they had no contact with the health services delivery points during the previous 12 months (i.e., neither visited a health facility nor were visited by a field worker at the community setting) (n=840). Thus, the included study population was 1,526 women, which corresponded to a weighted sample size of 1,650 women (**Figure 1**).

Variables

- ### Outcome variable

The outcome of this study is postpartum modern contraceptive uptake, dichotomised to "yes" or "no". Modern contraceptive uptake was defined when participants were using sterilisation (female or male), condoms (female or male), injectables, oral pills, intrauterine contraceptive device (IUCD), implants, lactational amenorrhea (LAM) and standard calendar method at the

For peer review only - http://bmjopen.bmj.com/site/about/guidelines.xhtml

time of the survey. Lack of uptake of a modern contraceptive method was defined as lack of use of any contraceptive method or traditional methods, such as abstaining and withdrawals [8].

- Exposure variable

The main exposure variable for this study was family planning counselling, which is defined as receiving family planning information either by a health service provider at a health facility or a field health worker within a community setting during the 12 months preceding the survey. We categorised the participants into two groups based on their exposure status. The first group consisted of participants who either visited a health facility or were visited by a field health worker in a community setting during the 12 months preceding the survey and received family planning information from a health worker during either of the two contact points were labelled as '*counselled*' for family planning. The second group consisted of participants who either visited a health facility or were visited by a field health worker in a community setting during the 12 months preceding the survey but did not receive family planning information from a health worker at either contact point were labelled as '*not counselled*' for family planning

- Covariates

After reviewing previous literature [9], we included sociodemographic characteristics, reproductive histories, and health service utilisation covariates for adjustment. The complete list of covariates used for adjustment and their definitions are included in the **supplementary material Table S1**.

Statistical analysis

We used a generalised linear regression model fitted with Poisson distribution and a log link function to examine the association between receipt of family planning counselling and postpartum modern contraceptive uptake after adjusting for sociodemographic characteristics, reproductive history, and health services utilisation. We applied two adjusted models. The first

For peer review only - http://bmjopen.bmj.com/site/about/guidelines.xhtml

model (*model 1*) adjusted for all covariates (**Table S1**). However, as the adjustment resulted in further loss of precision on the effect estimate, wide confidence intervals (CIs), we explored covariates that create a further loss of precision one by one and found to be a place of delivery, PNC visits, and postpartum period/ interview time. Later, we employed the second model (*model 2*), adjusting for all covariates, excluding a place of delivery, PNC, and postpartum period/interview time. We also run a sensitivity analysis to examine the association of family planning counselling and postpartum modern contraceptive uptake by stratifying the place of delivery, PNC visits and the postpartum period. The samples used in the main analysis and each stratified analysis were included in Table S2. The Chi-square test was used to check the distribution of covariates among counselled and non-counselled women. Multicollinearity among the covariate variables was checked using variance inflation factor (VIF). Unadjusted and adjusted relative risks (RRs) with 95% CIs were reported. All analyses accounted for the survey's non-response rate and complex sampling procedure and were undertaken with Stata 16 [24].

Patient and Public involvement

Patient or the public were not involved in this study

RESULTS

This study included 1,650 women who had contact with the health service during the 12 months preceding the EDHS survey (Figure 1). The characteristics of the study participants were included in Table S3. Less than half (48%) of the women reported that they received family planning counselling. Of the women who received counselling, 655 (82%) and 435 (55%) were rural residents and had no education. Of the counselled women, 648 (82%) had attended at least one ANC visit during their last pregnancy. However, 401 (52%) and 561 (72%) were women who gave birth at home and did not attend PNC visits, respectively. Except for maternal age, parity, distance to a health facility, health insurance coverage and receipt of media-

For peer review only - http://bmjopen.bmj.com/site/about/guidelines.xhtml

delivered family planning messages, all women's characteristics had similar distributions among those counselled and non-counselled. Of the counselled women, 361 (45%) were 30 years and above, whereas of the non-counselled women, 269 (21%) were 30 years and above. In reverse to this, of the counselled and non-counselled women, 231 (29%) and 318 (37%) were less than 25 years, respectively. Of those who received counselling from a health worker, 289 (36%) received family planning messages from the media, while of those who did not receive counselling from a health worker, only 208 (24%) received family planning messages from the media. Of the study women <6 months postpartum, 661 (84%) were postpartum amenorrhoeic, but only 473 (60%) met the LAM criteria. Indeed, more than three-quarters of women < 6 months postpartum in our study were not using modern contraceptive methods (**Table 1**).

Table 1. Characteristics of the study participants by the status of family planning counselling in Ethiopia 2016 (n=1,650)

**Variable**	**Category**	**Family planning counselling**	
		**Not counselled (%)**<br>**N=856 (51.9)**	**Counselled (%)**<br>**N= 794 (48.1)**
Household wealth index	Lowest	162 (18.9)	155 (19.6)
	Middle	379 (44.3)	306 (38.5)
	Highest	315 (36.8)	333 (41.9)
Residence	Urban	116 (13.5)	139 (17.5)
	Rural	740 (86.5)	655 (82.5)
Region	Tigray	67 (7.8)	93 (11.8)
	Amhara	189 (22.1)	174 (22.0)
	Oromia	344 (34.4)	299 (37.6)
	SNNP	165 (19.3)	175 (22.0)

For peer review only - http://bmjopen.bmj.com/site/about/guidelines.xhtml

For peer review only

	Addis Ababa	30 (3.5)	25 (3.2)
	Others	61 (7.2)	27 (3.5)
Women's age	<25 years	318 (37.2)	231 (29.1)
	25-29 years	269 (31.4)	202 (25.4)
	≥30 years	269 (21.4)	361 (45.5)
Women's educational level	No education	447 (52.2)	435 (54.8)
	Primary	314 (36.7)	271 (34.2)
	Secondary and above	95 (11.1)	87 (11.0)
Women's current job	Not working	542 (63.3)	454 (57.2)
	Working	314 (36.7)	340 (42.8)
Religion	Christian	477 (55.7)	505 (63.6)
	Muslim	362 (42.3)	273 (34.4)
	Others	17 (2.0)	16 (2.0)
Women's decision-making autonomy	No autonomy	308 (39.0)	262 (34.1)
	Having autonomy	481 (61.0)	506 (65.9)
Parity	Primiparity	240 (28.5)	165 (20.8)
	Multiparity	431 (50.4)	419 (52.8)
	Grand multiparity	181 (21.1)	210 (26.4)
Wantdeness of the last pregnancy	Unintended	232 (27.1)	217 (27.3)
	Intended	624 (72.9)	577 (72.7)
Sex of the last child	Male	419 (49.0)	402 (50.7)
	Female	437 (51.0)	391 (49.3)
Survival status of the last child	Died	20 (2.3)	21 (2.7)
	Alive	836 (97.7)	772 (97.3)

For peer review only - http://bmjopen.bmj.com/site/about/guidelines.xhtml

ANC visits for the last pregnancy	No	200 (23.4)	146 (18.4)
	Yes	654 (76.6)	648 (81.6)
Place of delivery for the last birth	Home	472 (56.2)	401 (51.6)
	Health facility	368 (43.8)	376 (48.4)
PNC visits following the last birth	No	642 (75.9)	561 (71.9)
	Yes	180 (24.1)	199 (28.1)
Interview time/ postpartum period	0-6 moths	468 (54.7)	466 (58.7)
	7-12 months	388 (45.3)	328 (41.3)
Fertility intention	No more	288 (33.8)	340 (43.0)
	Have another	565 (66.2)	451 (57.0)
Receiving family planning messages from media	No	648 (75.6)	505 (63.6)
	Yes	208 (24.4)	289 (36.4)
Have health insurance	No	834 (97.4)	737 (92.9)
	Yes	22 (2.6)	56 (7.1)
Distance to a health facility	A big problem	501 (58.6)	409 (51.6)
	Not a big problem	355 (41.4)	385 (48.4)

There were 445 (27%) participants who used modern contraceptive methods at the survey time. The most used modern contraceptive method was injectable (18.4%%), followed by implants (4.7%)**.** Two hundred forty-two (30%) and 204 (24%) of the counselled and not-counselled women used postpartum modern contraceptive methods, respectively (**Table 2**).

For peer review only - http://bmjopen.bmj.com/site/about/guidelines.xhtml

Table 2. The proportion of postpartum modern contraceptive use among counselled and not counselled women in Ethiopia 2016

Family planning counselling status	Postpartum modern contraceptive uptake	
	Yes (%)	No (%)
Counselled	242 (30)	553 (70)
Not counselled	204 (24)	652 (76)

Before adjustment, family planning counselling was associated with 27% higher postpartum modern contraceptive uptake (RR 1.27, 95% CI 1.00, 1.62). Moreover, the adjusted model (*model 1*) indicated that the risk of postpartum modern contraceptive uptake was 25% higher among women who have been counselled for family planning than those who did not receive counselling, although point estimates were imprecise (RR 1.32; 95% CI 1.04, 1.67). It was observed that place of delivery, PNC visits, and the postpartum period were the covariates that resulted in further loss of precision. The result of the adjusted model (*model 2*), excluding place of delivery, PNC visits, and postpartum period/interview time, also showed an association between family planning counselling and postpartum modern contraceptive uptake (RR 1.25; 95% CI 0.99, 1.60) (**Table 3**).

For peer review only - http://bmjopen.bmj.com/site/about/guidelines.xhtml

Table 3. Association between family planning counselling and postpartum modern contraceptive uptake stratified by place of delivery, PNC visits and interview time postpartum in Ethiopia 2016.

	**Unadjusted**	**Adjusted[1]**
All women	1.27 (1.00, 1.62)	1.25 (0.99, 1.60)
	Place of delivery	
Home delivery	1.35 (0.86, 2.13)	1.33 (0.83, 2.12)
Facility delivery	1.18 (0.90, 1.54)	1.27 (0.96, 1.67)
	PNC visits	
Had PNC visits	1.05 (0.73, 1.51)	1.25 (0.85, 1.83)
Had no PNC visits	1.39 (1.02, 1.90)	1.27 (0.92, 1.74)
	Interview time postpartum	
0-6 months postpartum	1.00 (0.66, 1.52)	0.90 (0.27, 2.96)
7-12 months postpartum	1.54 (1.17, 2.03)	1.60 (1.23, 2.07)

We performed a sensitivity analysis to further look at the effect of family planning counselling on postpartum contraceptive uptake by stratifying the place of delivery by home vs facility birth status, PNC visits as yes vs no, and the postpartum period into 0-6 vs 7-12 months. Although the effect estimates were imprecise (wide CIs), point estimates indicated that family planning counselling improved contraceptive uptake regardless of the place of delivery and PNC visits. However, the group for which counselling was observed to be associated with improved contraceptive uptake gave birth 7-12 months prior to undertaking the survey.

[1]Adjusted for household wealth status, residence, maternal age, maternal education, maternal employment, women's decision-making autonomy, religion, region, parity, survival status of the last-child, sex of last-child, wantdeness of last pregnancy, fertility intention, ANC , distance to health facility, health insurance coverage and receiving of family planning messages from media

For peer review only - http://bmjopen.bmj.com/site/about/guidelines.xhtml

DISCUSSION

This study evaluated the association between counselling for family planning and postpartum modern contraceptive uptake in Ethiopia using the EDHS 2016.

Our finding revealed that family planning counselling during the 12 months preceding the survey was associated with postpartum modern contraceptive uptake. This was supported by previous studies [15 20 21]. This was inconsistent with some previous studies from Ethiopia, which indicated a lack of association between family planning counselling and postpartum modern contraceptive uptake [22 25 26]. One reason for the inconsistency might be due to the differences in the quality of the family planning counselling. Although the previous studies conducted in Ethiopia on family planning counselling and postpartum contraceptive uptake did not describe the quality of the counselling, the quality can create a difference in adopting the contraceptive methods [27 28]. For example, a study conducted in Uttar Pradesh, India, showed that receiving higher quality family planning counselling improves the use of modern contraceptive methods [27]. Sociocultural differences might also explain the effect of family planning counselling on postpartum modern contraceptive uptake inconsistencies, as Ethiopia is multicultural with varied religious beliefs that may contribute to differences in perceptions and beliefs regarding contraceptive methods [28 29]. Therefore, even if women obtained counselling on family planning, this may not necessarily be translated to contraceptive use. Notable examples include sociocultural influences, such as gender inequality of power in decision-making or religious prohibitions to use contraceptive methods [28 30].

Our study also indicated that only half (48%) of the women received family planning counselling. This is slightly higher than the previous studies in Ethiopia, which indicated that only 28- 35% of women were provided family planning counselling [31 32]. This discrepancy might be due to the difference in family planning counselling definition. Unlike the previous studies that used only facility-based counselling, our definition includes counselling provided

For peer review only - http://bmjopen.bmj.com/site/about/guidelines.xhtml

in facility and/or community settings. Nonetheless, the result indicates that despite the fact that family planning services are integrated with maternal and child health services at the policy level [17 29], there remains a gap in the universal adoption of the recommendation by healthcare providers to counsel family planning, resulting in a higher proportion of women who missed the opportunity to be counselled. Although further research is needed on the barriers to the missed opportunity of providing family planning counselling while visiting service delivery points, previous qualitative research indicated that lack of awareness by the service providers of the national guidelines and the lack of the guidelines in the health facilities were the main barrier to not adhering on it [33]. The structural barriers, such as shortage of infrastructure and equipment and the unbalanced client-to-provider ratios within the Ethiopian health care system [34], might contribute to the high missed opportunity to provide family planning counselling.

Adjusted point estimates differed after additional adjustment for the place of delivery, PNC, and the postpartum period/interview time, implying that the pathway through which family planning counselling affects contraceptive uptake may act through these variables. We conducted additional sensitivity analyses to investigate further these pathways, which indicated that family planning counselling improved postpartum contraceptive uptake regardless of the place of delivery and PNC visits. However, we also observed that counselling was associated with improved postpartum contraceptive uptake only for women interviewed at least seven after giving birth. Delivery at a health facility or receipt of PNC that includes family planning counselling may not translate an increased likelihood of modern contraceptive uptake if women believe that they would be free of the risk of pregnancy while amenorrhoeic following delivery. Ethiopian women may consider resumption menses as critical timing for starting contraception in the postpartum period [26]. Ethiopian women may also believe that they are not at risk of pregnancy in the first six months after giving birth if they are breastfeeding their baby, whether or not breastfeeding is exclusive [35]. The findings of stronger associations between family

For peer review only - http://bmjopen.bmj.com/site/about/guidelines.xhtml

planning counselling and postpartum modern contraceptive uptake among women 7-12 months postpartum could also be because there might be a greater proportion of women who have re-engaged in sexual activity during this period than in the first six months.

Overall, our findings highlighted the importance of integration of family planning counselling with other health services, particularly with maternal and child health services. This helps dispel misperceptions and enable women to make informed decisions that would significantly reduce missed opportunities, thereby improving family planning counselling coverage and the uptake of postpartum modern contraception. Hence, the Ethiopian government and partner organisations with the remit of family planning, maternal and child health should work to translate the plans documented in the National Family Planning Guideline and the National Reproductive Health Strategy.

This is the first study that assessed the association between family planning counselling and postpartum modern contraceptive uptake at the national level and provides insight for government and partner organisations on the gaps in the provision of family planning counselling. However, this study has several limitations that need to be considered when interpreting our findings. The type and frequency of family planning information and the health worker who provided the counselling were not collected within the EDHS. Consequently, we were unable to examine the quality of counselling provided. Meanwhile, as the women were asked to provide family planning information for the 12 months prior to undertaking the survey, information before this time might have contributed to the association between family planning counselling and postpartum modern contraceptive uptake. Furthermore, although the data was based on self-reported information that occurred in the preceding 12 months, we cannot completely rule out the influence of recall bias. Lastly, due to the nature of the cross-sectional study design, it is not easy to determine the causal relationship between family planning counselling and postpartum modern contraceptive uptake.

For peer review only - http://bmjopen.bmj.com/site/about/guidelines.xhtml

CONCLUSION

Significant numbers of postpartum women did not receive family planning counselling despite contact with health service contact points during the preceding 12 months. We found that postpartum modern contraceptive uptake was low. Nonetheless, our findings reveal that family planning counselling is associated with improving postpartum modern contraceptive uptake in Ethiopia. Hence, besides preparing guidelines and working documents, the government should work to fully implement the integration of family planning counselling at all levels of service delivery points. Additionally, all service providers should deliver family planning counselling by complying with the national guideline, and postpartum counselling should stress the early initiation of modern contraceptives after giving birth. On top of these, continuous monitoring and evaluation of family planning counselling integration would help to reduce the missed opportunities and improve contraceptive uptake.

Author Contributions

Kalayu Brhane Mruts: Conceptualisation, Formal analysis, Data Curation, Writing- Original Draft, Writing-Review & Editing, Visualisation. **Gizachew A Tessema:** Writing-Review & Editing, Visualisation. **Jennifer Dunne**: Writing-Review & Editing, Visualisation. **Amanuel Tesfay Gebremedhin**: Writing-Review & Editing, Visualisation. **Jane A Scott**: Writing-Review & Editing, Visualisation. **Gavin Pereira**: Writing-Review & Editing, Visualisation

Ethical approval

The EDHs data was collected after ethical consent was sought from Science and Technology Ministry Ethiopia. Moreover, we obtained permission to use the EDHS dataset from the DHS program, which required no further ethical approval.

Patient consent for publication: not required

Data availability

Data are available in public open access in the DHS program repository

For peer review only - http://bmjopen.bmj.com/site/about/guidelines.xhtml

(https://dhsprogram.com/.). Registration is required to access the dataset.

Acknowledgments

We are grateful to the DHS program for providing access to the Ethiopian Demographic and Health Survey data

Declaration of conflicting interests

The authors have declared that no competing interests exist

Source of Funding

The authors did not receive financial or material support for this study from any public or private organisation. However, GP and GAT are investigators of Australian National Health and Medical Research Council Project Grants (#1099655 and #1173991 for GP, and #1195716 for GAT). GP also received institutional funding for the WA Health and Artificial Intelligence Consortium and is supported by the Research Council of Norway through its Centres of Excellence funding scheme #262700 for GP.

REFERENCES

1. Cleland J, Conde-Agudelo A, Peterson H, et al. Contraception and health. *Lancet* 2012;380(9837):149-56. doi: 10.1016/S0140-6736(12)60609-6 [published Online First: 2012/07/13]
2. Moore Z, Pfitzer A, Gubin R, et al. Missed opportunities for family planning: an analysis of pregnancy risk and contraceptive method use among postpartum women in 21 low- and middle-income countries. *Contraception* 2015;92(1):31-39. doi: 10.1016/j.contraception.2015.03.007
3. Ameyaw EK, Budu E, Sambah F, et al. Prevalence and determinants of unintended pregnancy in sub-Saharan Africa: A multi-country analysis of demographic and health surveys. *PloS one* 2019;14(8):e0220970-e70. doi: 10.1371/journal.pone.0220970
4. Bearak J, Popinchalk A, Ganatra B, et al. Unintended pregnancy and abortion by income, region, and the legal status of abortion: estimates from a comprehensive model for 1990–2019. *The Lancet Global Health* 2020;8(9):e1152-e61. doi: 10.1016/s2214-109x(20)30315-6
5. Mahande MJ, Obure J. Effect of interpregnancy interval on adverse pregnancy outcomes in northern Tanzania: a registry-based retrospective cohort study. *BMC Pregnancy Childbirth* 2016;16(1):140. doi: 10.1186/s12884-016-0929-5 [published Online First: 2016/06/09]
6. Rutstein SO. Effects of preceding birth intervals on neonatal, infant and under-five years mortality and nutritional status in developing countries: evidence from the demographic and health surveys. *International Journal of Gynecology & Obstetrics* 2005;89:S7-S24. doi: 10.1016/j.ijgo.2004.11.012

For peer review only - http://bmjopen.bmj.com/site/about/guidelines.xhtml

7. Tesema GA, Tessema ZT, Tamirat KS, et al. Prevalence of stillbirth and its associated factors in East Africa: generalized linear mixed modeling. *BMC Pregnancy Childbirth* 2021;21(1):414. doi: 10.1186/s12884-021-03883-6 [published Online First: 2021/06/04]
8. Central Statistical Agency - CSA/Ethiopia, ICF. Ethiopia Demographic and Health Survey 2016. Addis Ababa, Ethiopia: CSA and ICF, 2017.
9. Dagnew GW, Asresie MB, Fekadu GA, et al. Modern contraceptive use and factors associated with use among postpartum women in Ethiopia; further analysis of the 2016 Ethiopia demographic and health survey data. *BMC Public Health* 2020;20(1):661. doi: 10.1186/s12889-020-08802-6
10. Mengesha ZB, Worku AG, Feleke SA. Contraceptive adoption in the extended postpartum period is low in Northwest Ethiopia. *BMC Pregnancy and Childbirth* 2015;15(1):160. doi: 10.1186/s12884-015-0598-9
11. Teshale AB, Tesema GA. Magnitude and associated factors of unintended pregnancy in Ethiopia: a multilevel analysis using 2016 EDHS data. *BMC Pregnancy and Childbirth* 2020;20(1) doi: 10.1186/s12884-020-03024-5
12. Endalamaw A, Engeda EH, Ekubagewargies DT, et al. Low birth weight and its associated factors in Ethiopia: a systematic review and meta-analysis. *Ital J Pediatr* 2018;44(1):141. doi: 10.1186/s13052-018-0586-6 [published Online First: 2018/11/28]
13. Jena BH, Biks GA, Gelaye KA, et al. Magnitude and trend of perinatal mortality and its relationship with inter-pregnancy interval in Ethiopia: a systematic review and meta-analysis. *BMC Pregnancy and Childbirth* 2020;20(1) doi: 10.1186/s12884-020-03089-2
14. Muchie KF, Lakew AM, Teshome DF, et al. Epidemiology of preterm birth in Ethiopia: systematic review and meta-analysis. *BMC Pregnancy Childbirth* 2020;20(1):574. doi: 10.1186/s12884-020-03271-6 [published Online First: 2020/10/01]
15. Amour C, Manongi RN, Mahande MJ, et al. Missed opportunity for family planning counselling along the continuum of care in Arusha region, Tanzania. *PLOS ONE* 2021;16(7):e0250988. doi: 10.1371/journal.pone.0250988
16. World Health Organization. Programming strategies for postpartum family planning, 2013.
17. Federal Democratic Republic of Ethiopia MoH. National Guideline for Family Planning Services in Ethiopia, 2020.
18. Dehlendorf C, Krajewski C, Borrero S. Contraceptive counseling: best practices to ensure quality communication and enable effective contraceptive use. *Clinical obstetrics and gynecology* 2014;57(4):659.
19. Zapata LB, Murtaza S, Whiteman MK, et al. Contraceptive counseling and postpartum contraceptive use. *American journal of obstetrics and gynecology* 2015;212(2):171. e1-71. e8.
20. Tafa L, Worku Y. Family planning utilization and associated factors among postpartum women in Addis Ababa, Ethiopia, 2018. *PLoS One* 2021;16(1):e0245123. doi: 10.1371/journal.pone.0245123 [published Online First: 2021/01/23]
21. Tafere TE, Afework MF, Yalew AW. Counseling on family planning during ANC service increases the likelihood of postpartum family planning use in Bahir Dar City Administration, Northwest Ethiopia: a prospective follow up study. *Contracept Reprod Med* 2018;3:28. doi: 10.1186/s40834-018-0081-x [published Online First: 2019/01/05]
22. Woldu BF, Ermolo TL, Lemu LG, et al. Long-acting reversible contraception utilization and associated factors among women in extended postpartum period in Hossana town, southern Ethiopia: cross sectional study. *Contracept Reprod Med* 2020;5(1):10. doi: 10.1186/s40834-020-00117-6 [published Online First: 2020/08/11]
23. Zimmerman LA, Yi Y, Yihdego M, et al. Effect of integrating maternal health services and family planning services on postpartum family planning behavior in Ethiopia: results from a longitudinal survey. *BMC Public Health* 2019;19(1):1448. doi: 10.1186/s12889-019-7703-3 [published Online First: 2019/11/07]

For peer review only - http://bmjopen.bmj.com/site/about/guidelines.xhtml

24. StataCorp L. Stata Statistical Software. Release 16.[software]. College Station, TX: Stata Press. Available at: https://www. stata. com/. Accessed September, 2019.
25. Gebremariam A, Gebremariam H. Contraceptive use among lactating women in Ganta-Afeshum District, Eastern Tigray, Northern Ethiopia, 2015: a cross sectional study. *BMC Pregnancy Childbirth* 2017;17(1):421. doi: 10.1186/s12884-017-1613-0 [published Online First: 2017/12/17]
26. Gejo NG, Anshebo AA, Dinsa LH. Postpartum modern contraceptive use and associated factors in Hossana town. *PLoS One* 2019;14(5):e0217167. doi: 10.1371/journal.pone.0217167 [published Online First: 2019/05/23]
27. Dehingia N, Dixit A, Averbach S, et al. Family planning counseling and its associations with modern contraceptive use, initiation, and continuation in rural Uttar Pradesh, India. *Reprod Health* 2019;16(1):178. doi: 10.1186/s12978-019-0844-0 [published Online First: 2019/12/14]
28. Edberg M, Sedlander E, Rimal R, et al. Planned social network change and modern contraceptive use in a rural Ethiopian community. *Journal of Global Health Reports* 2018;2 doi: 10.29392/joghr.2.e2018034
29. Ethiopia Federal Ministry of Health. National Reproductive Health Strategy 2016-2020: Federal Ministry of Health (FMOH) Addis Ababa, Ethiopia, 2016.
30. Tigabu S, Demelew T, Seid A, et al. Socioeconomic and religious differentials in contraceptive uptake in western Ethiopia: a mixed-methods phenomenological study. *BMC Women's Health* 2018;18(1) doi: 10.1186/s12905-018-0580-6
31. Teshome A, Birara M, Rominski SD. Quality of family planning counseling among women attending prenatal care at a hospital in Addis Ababa, Ethiopia. *International Journal of Gynecology and Obstetrics* 2017;137(2):174-79. doi: 10.1002/ijgo.12110
32. Abdulreshid M, Dadi HB. Assessment of Family Planning Counseling Provided for Postpartum Women and Associated Factors. *International Journal of Reproductive Medicine* 2020;2020:2649340. doi: 10.1155/2020/2649340
33. Tessema G. A. Health provider perspectives on implementation of family planning guidelines in family planning services. The University of Adelaide, 2018.
34. Assefa Y, Gelaw YA, Hill PS, et al. Community health extension program of Ethiopia, 2003-2018: successes and challenges toward universal coverage for primary healthcare services. *Global Health* 2019;15(1):24. doi: 10.1186/s12992-019-0470-1 [published Online First: 2019/03/28]
35. Labbok MH, Hight-Laukaran V, Peterson AE, et al. Multicenter study of the Lactational Amenorrhea Method (LAM): I. Efficacy, duration, and implications for clinical application. *Contraception* 1997;55(6):327-36. doi: 10.1016/s0010-7824(97)00040-1 [published Online First: 1997/06/01]

For peer review only

For peer review only - http://bmjopen.bmj.com/site/about/guidelines.xhtml

Figure 1. Participant's selection

Note: The sample weights account for the probability of selection and interview in the EDHS 2016.

For peer review only

For peer review only - http://bmjopen.bmj.com/site/about/guidelines.xhtml

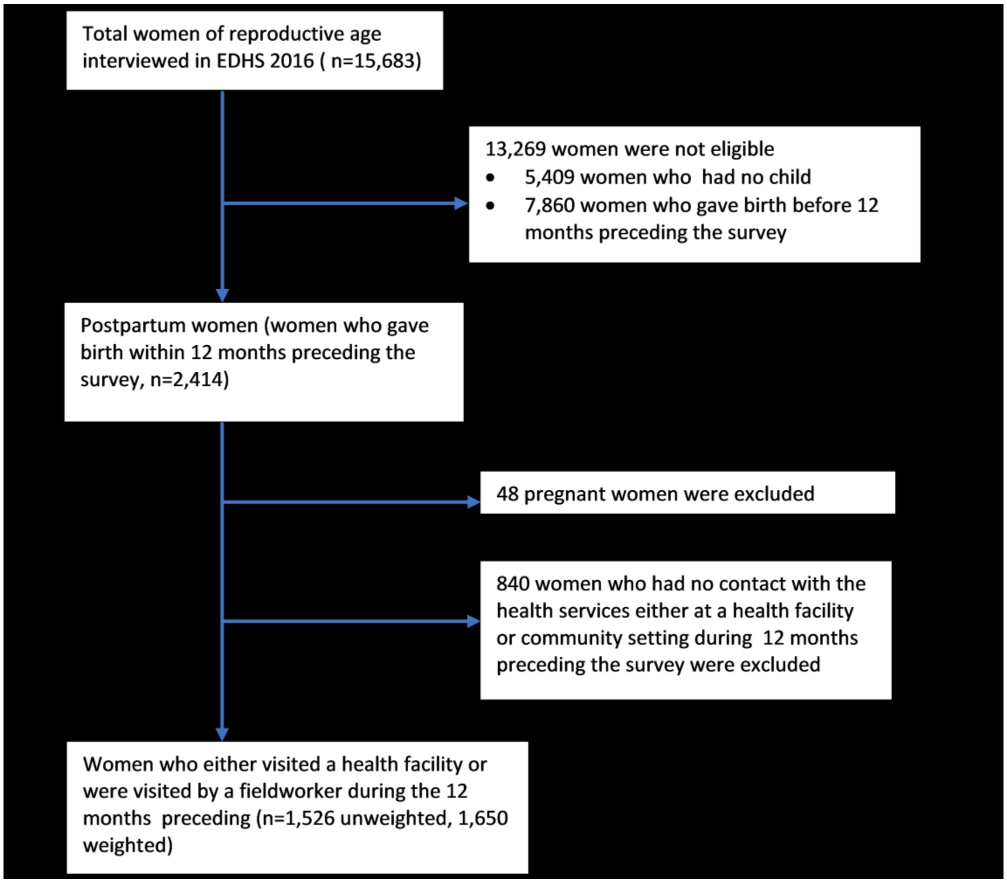

Figure 1. Participant's selection

665x581mm (144 x 144 DPI)

Table S1; List of covariates and their definition

Variable	Definition
Household wealth quintile	The household wealth index was computed by principal component analysis (PCA) using the household's ownership of selected items, such as radio and television, and possession of household animals, including oxen, cow, camel; and materials used for housing construction, and types of water access and sanitation facilities taking into consideration of urban-rural difference. We categorised the wealth index score into three equal parts labelled as lowest, middle, and highest
Residence	Participant place of residence coded as urban and rural. Urban was defined as a locality with 2,000 or more inhabitants. Moreover, all administrative capitals, including Region, Zone and Woreda, and localities in which urban dwellers' associations were established were considered urban residences, irrespective of the population size. Rural residences comprise all areas not classified as urban.
Region	Ethiopia has nine regional states and two city administrations. For this study purpose, we merged the emerging regional states of Ethiopia, such as Afar, Somali, Benishangul-Gumuz, Harari and Dire Dawa, into one group as others. Hence, the region categorised into Tigray, Amhara, Oromia, SNNP, Addis Ababa and others
Women's age	Women's age was grouped into <25 years, 25-29 years, and ≥30 years
Women's educational status	Women's education status is available in EDHS coded as no education, primary, secondary, and higher. Since the frequency of women who had a higher level of education was low, we merged the secondary and higher into 'secondary and above.'
Women's employment	Participants were grouped as employed when they reported as working within seven days preceding the survey, including women who did not work in the past seven days but who are regularly employed and were absent from work for leave, illness, vacation, or any other such reason, otherwise as not employed
Marital status	The marital status was categorised as married/in union, including married and living with a partner, and not married/in union, including never in union, widowed, divorced, and no longer living together/separated
Women's religion	Women's religion is available in the EDHS coded as Orthodox, Muslim, Protestant, Catholic, Traditional, and other. We merged Orthodox, Protestant and Catholic into Christian and Traditional and

For peer review only

	others into other. Hence, religion in this study was categorised as Christian, Muslim and Others
Parity	Parity of the participant women was categorised into primiparity (para 1), multiparity (para 2-4) and grand multiparity (para ≥5)
Wantdeness of the last child	Wantdeness of the last child grouped into planned, unplanned, and unwanted
Sex of last-child	Sex of the last child was categorised as male or female
Survival status of the last child	Survival status of the last child categorised as dead and alive
ANC for the pregnancy of the last birth	ANC visit at least once during the pregnancy of last birth was coded as yes, if not as no
Place of delivery for the last birth	Place of delivery was labelled as home if delivered at their home or any other home, and health facility if delivered in any one of the health facilities- public, private, or non-governmental health facility.
PNC following the last birth	PNC was categorised as yes when they received any checking their health status either at a health facility or at home within six weeks following the last birth; otherwise, no
Postpartum period/interview time	The postpartum period is the time between the birth of the last child and the interview date and was categorised into 0-6 months and 7-12 months
Future fertility intention	Future fertility intention was categorised as have more, no more and undecided
Receiving family planning messages from media	receiving of family planning messages from media was labelled as 'yes' when women received any family planning messages from any one of the following: radio, television, newspaper/magazine or text message on mobile phone in the past few months preceding the survey, and 'no' if did not receive any message from any one of the media channels.
Distance to a health facility for getting medical help	The distance to a health facility for getting medical help was captured by a self-reported response as a big problem vs not a big problem
Health insurance	Health insurance was categorised as yes and no, corresponding for having and not having health insurance, respectively.

For peer review only

Table S2. Sample size for the adjusted and unadjusted models among all and women stratified by place of delivery, PNC visits and interview time postpartum in Ethiopia 2016

Samples		**Unadjusted**	**Adjusted**
All women		1,650	1,550
Place of delivery	Home delivery	873	831
	Facility delivery	743	689
PNC visits	Had PNC visits	423	394
	Had no PNC visits	1,203	1,134
Interview time postpartum	0-6 months postpartum	934	888
	7-12 months postpartum	716	662

For peer review only

Table S3. Sociodemographic characteristics, reproductive histories, and health service utilisations of the study participants in Ethiopia 2016 (n=1,650)

Variable	Category	Frequency	Percentage
Household wealth index	Lowest	317	19.2
	Middle	685	41.5
	Highest	647	39.2
Residence	Urban	255	15.4
	Rural	1,395	84.6
Region	Tigray	160	9.7
	Amhara	363	22.0
	Oromia	642	38.9
	SNNP	340	20.6
	Addis Ababa	55	3.3
	Others	89	5.4
Women's age at birth of the last child	<25 years	634	38.4
	25-29 years	456	27.7
	≥30 years	559	33.9
Women's educational level	No education	881	53.4
	Primary	586	35.5
	Secondary and above	183	11.1
Marital status	Not married/in union	73	4.4
	Married/in union	1,577	95.6
Women's current job	Not working	996	60.4
	Working	654	39.6
Religion	Christian	981	59.5
	Muslim	635	38.5
	Others	33	2.0
Women's decision-making autonomy	No autonomy	570	36.6
	Having autonomy	987	63.4
Parity	Primiparity	409	24.8
	Multiparity	850	51.5
	Grand multiparity	390	23.7
Wantdeness of the last pregnancy	Unintended	449	27.2
	Intended	1,200	72.8
Sex of the last child	Male	822	49.8
	Female	828	50.2
Survival status of the last child	Died	41	2.5
	Alive	1,608	97.5
ANC visits for pregnancy of the last birth	No	346	21.0
	Yes	1,302	79.0
Place of delivery for the last birth	Home	873	54.0
	Health facility	744	46.0
PNC visits following the last birth	No	1,203	76.1
	Yes	379	23.9
Postpartum period/ Interview time	0-6 moths	934	56.6
	7-12 months	716	43.4
Fertility intention	No more	629	38.2
	Have another	1,017	61.8
receiving of family planning messages from media	No	1,152	69.8
	Yes	498	30.2
Distance to a health facility	A big problem	911	55.2
	Not a big problem	739	44.8
Have health insurance	No	1,571	95.2
	Yes	78	4.8
Family planning counselling	Not counselled	856	51.9
	Counselled	794	48.1

For peer review only

Reporting checklist for cross sectional study.

Based on the STROBE cross sectional guidelines.

Instructions to authors

Complete this checklist by entering the page numbers from your manuscript where readers will find each of the items listed below.

Your article may not currently address all the items on the checklist. Please modify your text to include the missing information. If you are certain that an item does not apply, please write "n/a" and provide a short explanation.

Upload your completed checklist as an extra file when you submit to a journal.

In your methods section, say that you used the STROBE cross sectionalreporting guidelines, and cite them as:

von Elm E, Altman DG, Egger M, Pocock SJ, Gotzsche PC, Vandenbroucke JP. The Strengthening the Reporting of Observational Studies in Epidemiology (STROBE) Statement: guidelines for reporting observational studies.

		Reporting Item	Page Number
**Title and abstract**			
Title	#1a	Indicate the study’s design with a commonly used term in the title or the abstract	1

For peer review only

Abstract	#1b	Provide in the abstract an informative and balanced summary of what was done and what was found	2
**Introduction**			
Background / rationale	#2	Explain the scientific background and rationale for the investigation being reported	5-6
Objectives	#3	State specific objectives, including any prespecified hypotheses	6
**Methods**			
Study design	#4	Present key elements of study design early in the paper	7
Setting	#5	Describe the setting, locations, and relevant dates, including periods of recruitment, exposure, follow-up, and data collection	7
Eligibility criteria	#6a	Give the eligibility criteria, and the sources and methods of selection of participants.	7
	#7	Clearly define all outcomes, exposures, predictors, potential confounders, and effect modifiers. Give diagnostic criteria, if applicable	7-8
Data sources / measurement	#8	For each variable of interest give sources of data and details of methods of assessment (measurement). Describe comparability of assessment methods if there is more than one group. Give information separately for for exposed and unexposed groups if applicable.	7-8

For peer review only

Bias	#9	Describe any efforts to address potential sources of bias	8-9
Study size	#10	Explain how the study size was arrived at	7
Quantitative variables	#11	Explain how quantitative variables were handled in the analyses. If applicable, describe which groupings were chosen, and why	8-9
Statistical methods	#12a	Describe all statistical methods, including those used to control for confounding	8-9
Statistical methods	#12b	Describe any methods used to examine subgroups and interactions	9
Statistical methods	#12c	Explain how missing data were addressed	n/a
Statistical methods	#12d	If applicable, describe analytical methods taking account of sampling strategy	9
Statistical methods	#12e	Describe any sensitivity analyses	9

Results

Participants	#13a	Report numbers of individuals at each stage of study—eg numbers potentially eligible, examined for eligibility, confirmed eligible, included in the study, completing follow-up, and analysed. Give information separately for for exposed and unexposed groups if applicable.	9
Participants	#13b	Give reasons for non-participation at each stage	9

For peer review only

Participants	#13c	Consider use of a flow diagram	9
Descriptive data	#14a	Give characteristics of study participants (eg demographic, clinical, social) and information on exposures and potential confounders. Give information separately for exposed and unexposed groups if applicable.	9
Descriptive data	#14b	Indicate number of participants with missing data for each variable of interest	9
Outcome data	#15	Report numbers of outcome events or summary measures. Give information separately for exposed and unexposed groups if applicable.	9-12
Main results	#16a	Give unadjusted estimates and, if applicable, confounder-adjusted estimates and their precision (eg, 95% confidence interval). Make clear which confounders were adjusted for and why they were included	13-14
Main results	#16b	Report category boundaries when continuous variables were categorized	n/a
Main results	#16c	If relevant, consider translating estimates of relative risk into absolute risk for a meaningful time period	n/a
Other analyses	#17	Report other analyses done—e.g., analyses of subgroups and interactions, and sensitivity analyses	13-14
**Discussion**			
Key results	#18	Summarise key results with reference to study objectives	15

For peer review only

Limitations	#19	Discuss limitations of the study, taking into account sources of potential bias or imprecision. Discuss both direction and magnitude of any potential bias.	17
Interpretation	#20	Give a cautious overall interpretation considering objectives, limitations, multiplicity of analyses, results from similar studies, and other relevant evidence.	15-17
Generalisability	#21	Discuss the generalisability (external validity) of the study results	17
**Other Information**			
Funding	#22	Give the source of funding and the role of the funders for the present study and, if applicable, for the original study on which the present article is based	19

The STROBE checklist is distributed under the terms of the Creative Commons Attribution License CC-BY. This checklist was completed on 15. December 2021 using https://www.goodreports.org/, a tool made by the EQUATOR Network in collaboration with Penelope.ai

For peer review only
